# *Leptospira* Immunoglobulin-Like Protein B Interacts with the 20th Exon of Human Tropoelastin Contributing to *Leptospiral* Adhesion to Human Lung Cells

**DOI:** 10.3389/fcimb.2017.00163

**Published:** 2017-05-09

**Authors:** Ching-Lin Hsieh, Andrew Tseng, Hongxuan He, Chih-Jung Kuo, Xuannian Wang, Yung-Fu Chang

**Affiliations:** ^1^Department of Population Medicine and Diagnostic Sciences, College of Veterinary Medicine, Cornell UniversityIthaca, NY, USA; ^2^National Research Center for Wildlife Borne Diseases, Institute of Zoology, Chinese Academy of SciencesBeijing, China; ^3^Department of Veterinary Medicine, National Chung Hsing UniversityTaichung, Taiwan; ^4^Research Center for Biotechnology, Xinxiang UniversityXinxiang, China

**Keywords:** LigB, *Leptospira*, tropoelastin, outer surface protein, extracellular matrix proteins, protein-protein interaction

## Abstract

*Leptospira* immunoglobulin-like protein B (LigB), a surface adhesin, is capable of mediating the attachment of pathogenic *leptospira* to the host through interaction with various components of the extracellular matrix (ECM). Human tropoelastin (HTE), the building block of elastin, confers resilience and elasticity to lung, and other tissues. Previously identified Ig-like domains of LigB, including LigB4 and LigB12, bind to HTE, which is likely to promote *Leptospira* adhesion to lung tissue. However, the molecular mechanism that mediates the LigB-HTE interaction is unclear. In this study, the LigB-binding site on HTE was further pinpointed to a N-terminal region of the 20th exon of HTE (HTE20N). Alanine mutants of basic and aromatic residues on HTE20N significantly reduced binding to the LigB. Additionally, HTE-binding site was narrowed down to the first β-sheet of LigB12. On this binding surface, residues F1054, D1061, A1065, and D1066 were critical for the association with HTE. Most importantly, the recombinant HTE truncates could diminish the binding of LigB to human lung fibroblasts (WI-38) by 68%, and could block the association of LigA-expressing *L. biflexa* to lung cells by 61%. These findings should expand our understanding of leptospiral pathogenesis, particularly in pulmonary manifestations of leptospirosis.

## Introduction

Leptospirosis, a neglected yet serious infectious disease caused by pathogenic *Leptospira*, is considered the most widespread zoonosis in the world. Both humans and animals could contract leptospirosis by exposing eroded skin or mucosa to spirochete contaminated water or soils (Meites et al., 2004; Palaniappan et al., 2007). After entering host circulation system, the bacteria can rapidly disseminate throughout the body and then reach the target organs such as liver, lung, kidney, leading to devastating multi-organ failures, also known as Weil's disease (Ko et al., 2009; Haake and Levett, 2015). In fatal cases of leptospirosis, pulmonary hemorrhage has been thought to be a major lethal factor (Levett, 2001; Taylor et al., 2015).

To establish the initial step of infection, pathogenic bacteria adhere to host tissues by utilizing microbial surface components recognizing adhesive matrix molecules (MSCRAMMs) to interact with host extracellular matrix components (ECM) (Patti et al., 1994). To date, a large number of leptospiral MSCRAMMs have been identified and thought to play multiple roles in binding to host ECM (Lin and Chang, 2007; Stevenson et al., 2007; Lin et al., 2009, 2010; Pinne et al., 2010). Among those MSCRAMMs, the *Leptospira* immunoglobulin-like (Lig) protein family is exclusively presented on the outer membrane of pathogenic *Leptospira*. The family contains three paralogs of proteins LigA, LigB, and LigC, which, respectively, consist of 13, 12, and 13 immunoglobulin-like (Ig-like) domains. The N-terminal six domains and half of seventh domain of LigA (amino acids 1–630) shares exactly identical sequences with LigB, while the remaining C-terminal domains of two Lig proteins are distinct. LigC is a pseudogene in many strains, suggesting its minor role as a virulence factor (Palaniappan et al., 2002; Matsunaga et al., 2003). Interestingly, each Ig-like domain was found to have diverse host binding partners and to likely participate in different stages of bacterial attachment to host tissues. For example, the 4th domain (LigB4) and the 12th domain (LigB12) of LigB bind to human tropoelastin (Lin et al., 2009), and LigB12 also recognizes fibrinogen and fibronectin (Lin et al., 2010, 2011). Although *L. interrogans* ligB mutant could still adhere to canine kidney cells, it is likely that LigA complements the adhesion role that LigB was thought to play (Croda et al., 2008). Furthermore, expressing Lig proteins on the surface of *L. biflexa* allowed the non-pathogenic spirochetes to gain the ability to bind to ECM molecules and to associate with mammalian cells (Figueira et al., 2011), which suggests Lig proteins are important for bacteria-host interactions.

Elastin, one of the major components of ECM, is predominately abundant in lung, skin, major arteries, uterus, and placenta (Graf et al., 1996; Mithieux and Weiss, 2005). Given the inherent elasticity and resilience of elastin, these tissues were able to maintain the structural integrity during the process of periodic distension. Tropoelastin, the building block of the elastin, is composed of alternating hydrophobic domains and crosslinking domains. Through the coacervation and cross-linking processes, the tropoelastin monomers associate with each other to form elastin (Nivison-Smith and Weiss, 2011). Because of the universal prevalence of elastin on the mammalian cell surface, bacterial pathogens have developed several MSCRAMMs to recognize elastin in order to establish the infection (Keane et al., 2007a,b; Kuo et al., 2013). For pathogenic *Leptospira*, we firstly identified that Ig-like domains of LigB were responsible for binding to the central region of human tropoelastin (HTE) (Lin et al., 2009). Other groups also found that Omp37 and Omp47 had similar roles to Lig proteins (Pinne et al., 2010). However, the detailed binding mechanism and the key LigB-interacting residues of HTE have not yet been revealed.

To this end, we pinpointed the critical binding residues for HTE-LigB interaction. The LigB minimal binding site was narrowed down to N-terminal half of HTE20 (HTE20N) where the arginine and the aromatic residues were required for binding to LigB. HTE20N interacted with the first β-strand of LigB12 where F1054, D1061, A1065, and D1066 were important for the LigB-HTE interaction. Finally, the recombinant HTE truncates could diminish the binding of LigB to human lung fibroblasts (WI-38 cells) by 68%, and could block the association of Lig-expressing spirochetes to WI-38 cells by 61%.

## Materials and methods

### Bacterial strains and cell culture

*Leptospira biflexa* serovar Patoc *ligA*, a generous gift from Dr. Albert I Ko (Figueira et al., 2011), was grown in Ellinghausen-McCullough-Johnson-Harris (EMJH) medium at 30°C. Because *L. biflexa* serovar Patoc *ligB* was unable to express intact LigB, we used *L. biflexa* serovar Patoc *ligA* instead (Figueira et al., 2011). The human embryonic lung fibroblasts, WI-38 cells (ATCC CCL-75), were cultured in Eagle's minimum essential medium (EMEM) supplemented with 10% fetal bovine serum (FBS) (GIBCO) and were grown at 37°C in a humidified atmosphere with 5% CO_2_. *Escherichia coli* TOP10 (Invitrogen) and Rosetta (DE3) strains (Novagen) were cultured in Luria-Bertani broth (LB) with appropriate antibiotics at 37°C.

### Reagents and antibodies

Tropoelastin (purified from chicken aorta) was purchased from Elastin Product Co. (Owensville, MO). Sensor chip CM5, sodium acetate buffer, 1-ethyl-3-(3-dimethylaminopropyl)-carbodiimide (EDC) and N-hydroxysuccinimide (NHS), ethanolamine and glycine-HCl were purchased from GE Healthcare (Marlborough, MA). Rabbit anti-GST IgG antibodies conjugated with HRP was purchased from GenScript (Piscataway, NJ). HRP-conjugated goat anti-hamster IgG antibody and 3,3′,5,5′-tetramethylbenzidine (TMB) peroxidase substrate were purchased from Kirkegaard and Perry Laboratories (Gaithersburg, MD). Polyclonal antibodies specifically against *L. biflexa* serovar Patoc *ligA* were generated by immunizing hamsters with the same spirochetes twice, and the anti-sera were collected from hamsters 1 week after second immunization. Hamsters were used under conformity of animal protocols, which were approved by Cornell University Institutional Animal Care and Use Committee (IACUC, Protocol number: 2015-0133). Animals were cared for in adherence to the policies of the NIH Office of Laboratory Animal Welfare (OLAW), the standards of the Animal Welfare Act, the Public Health Service Policy, and the Guide for the Care and Use of Laboratory Animals.

### Plasmid construction and protein purification

**Figure 1:**
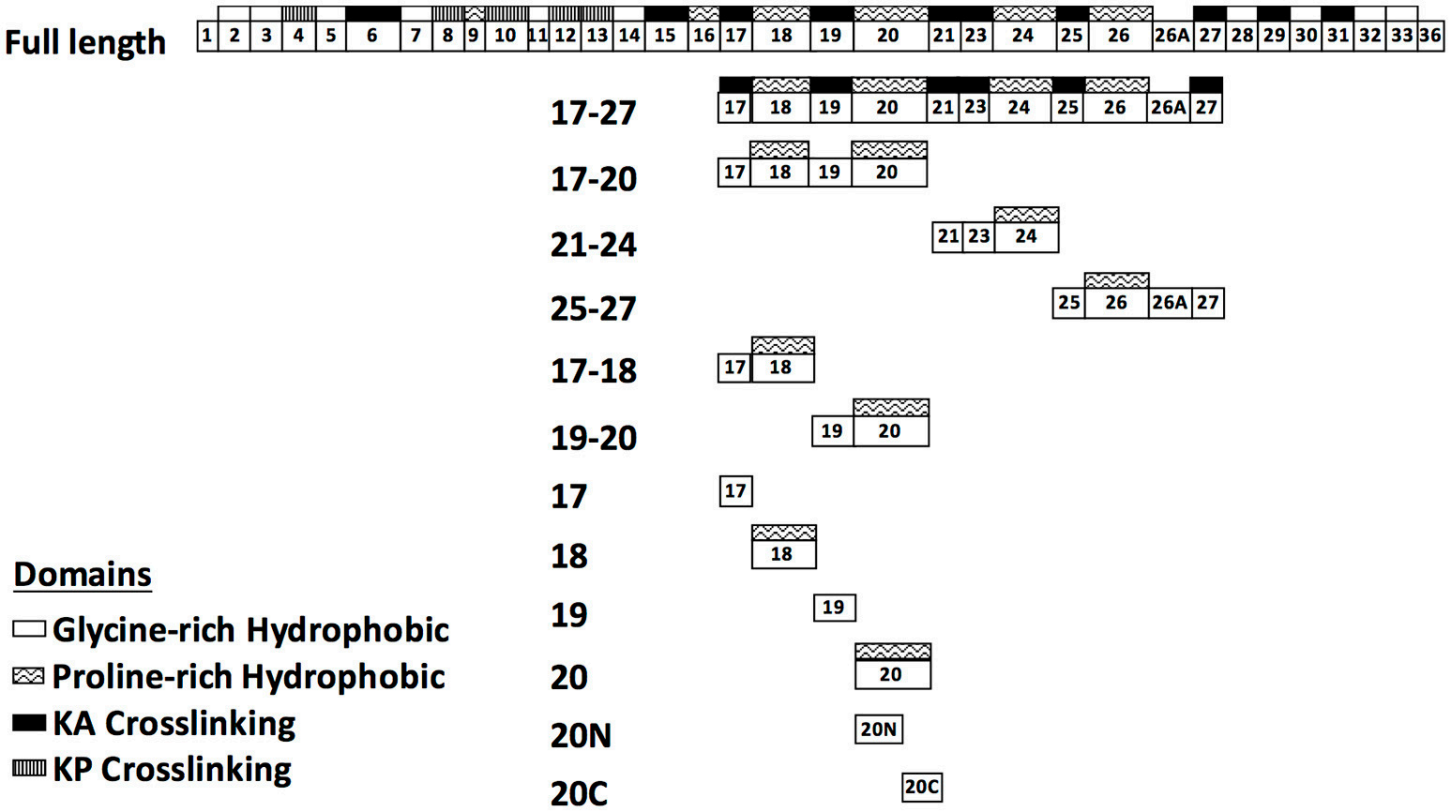
**Schematic representation of human tropoelastin (HTE) and the truncated constructs used in this study**. HTE is composed of alternating hydrophobic domains (glycine-rich or proline-rich domain) and crosslinking domains (KA or KP crosslinking domain). The truncated HTE used in this study are indicated.

Truncated LigB genes, LigB4 (amino acids 307–403 in LigB) and LigB12 (amino acids 1,047–1,119 in LigB) were amplified by PCR based on the DNA sequences derived from GenBankTM (*L. interrogans* serovar Pomona, FJ030916) and further constructed into pGEX-6P-1 and pGEX-4T-2 vector (GE Healthcare) to express as GST-tagged proteins. LigB4, LigB5, LigB7, LigB10, and LigB12 were also subcloned into pET28-SUMO vector as previously described to express as His-Sumo tagged proteins (Manford et al., 2010). A series of human tropoelastin (HTE) truncates, as shown in Figure 1, including HTE17-27 (17th to 27th exon of HTE), HTE17-20, HTE21-24, HTE25-27, HTE17-18, HTE19-20, HTE17, HTE18, HTE19, and HTE20 were amplified by PCR using the primers listed in Supplementary Table 1 and using the construct HTE17-27/pGEX-4T-2 as a template (Lin et al., 2009). Similarly, HTE20N (the first 27 residues of HTE20, amino acids 358–384 of HTE) and HTE20C (the last 28 residues of HTE20, amino acids 385–412 of HTE) truncates were generated using the primers listed in Supplementary Table 1. All amplified HTE fragments were digested with EcoRI and XhoI, and then inserted into pET28-SUMO cut with the same restriction enzymes. Following the manufacturer's instruction of QuickChange mutagenesis kit (Stratagene), four HTE20 mutants (R360A, Y371A, F378A, and F381A) were generated by using wild-type HTE20/pET28-SUMO as a template and corresponding primers addressed in Supplementary Table 2. Likewise, five LigB12 mutants (F1054A, D1061N, A1065K, D1066A, and E1088A) were generated by using wild-type LigB12/pET28-SUMO as a template and by using the primers listed in Supplementary Table 2. The sequence-confirmed constructs were then, respectively, transformed into *E. coli* Rosetta strains for protein expressions. For GST tagged LigB4 and LigB12, the glutathione agaroses pre-equilibrated with PBS buffer (pH = 7.5) were used for purification as previously described (Kuo et al., 2013). Additionally, His-SUMO tagged HTE truncations and Lig proteins were purified by the Ni^2+^-NTA resins. Low concentrations (10 and 30 mM) of imidazole solutions were used to remove the unwanted proteins from Ni^2+^-NTA resins. Then, 300 mM of imidazole solutions were used to elute His-SUMO tagged proteins. The eluate was dialyzed against PBS buffer. Concurrently, His-SUMO tag was digested with SUMO-specific protease Ulp-1 at 4°C overnight. A second Ni^2+^-NTA column was further used for His-SUMO tag removal. The tag free proteins were finally applied to size exclusion chromatography (HiLoad 16/600 Superdex 75) to gain higher purity for the following experiments.

### Generating LigB5/LigB12 and LigB7/LigB10 chimeric constructs

To generate a full set of LigB5/LigB12 and LigB7/LigB10 chimeric constructs, a series of overlapping extension PCR were conducted by using the primers listed in Supplementary Table 3. Generally, each single domain was further divided into three segments, strand A–C, strand C'–F, and strand G–G'. The strand boundaries were rationally determined based on the spatial arrangement of β-sheets in the high-resolution structure of LigB12 (Ptak et al., 2014). In the first run of the constructions, either one or two consecutive segments at N terminus or C terminus were swapped with corresponding segments from the other Ig-like domain, generating the chimeric amplicons LigB5-LigB5-LigB12, LigB5-LigB12-LigB12, LigB12-LigB12-LigB5 and LigB12-LigB5-LigB5, LigB7-LigB7-LigB10, LigB7-LigB10-LigB10, LigB10-LigB10-LigB7 and LigB10-LigB7-LigB7. Subsequently, the central segments were swapped in the next run of the constructions, producing LigB5-LigB12-LigB5, LigB12-LigB5-LigB12, LigB7-LigB10-LigB7 and LigB10-LigB7-LigB10. BamHI and HindIII (or XhoI) restriction enzyme sites were artificially introduced to 5′ and 3′ end of all PCR amplicons to facilitate the ligations into pET28-Sumo vectors.

### Binding assays by ELISA

To examine the binding affinity of HTE truncates to Lig proteins, 1 μM of various HTE fragments were coated on microtiter wells, respectively, in 0.1 M of NaHCO_3_ (pH 9.4) coating buffer. HTE17-27 (Lin et al., 2009) and BSA were included in the binding assay as a positive and a negative control, respectively. To investigate the critical LigB interacting residues of HTE20, different HTE20 mutants R360A, Y371A, F378A, and F381A were also individually immobilized on the microtiter wells using the same condition addressed above. All microtiter plates were blocked with PBS buffer containing 3% BSA, and then a series 2-fold dilutions of GST tagged Lig proteins (10 to 0.156 μM) were applied to HTE truncates coated wells for 1 h at 37°C. Subsequently, HRP-conjugated rabbit anti-GST IgG antibodies (1:2,000) were added to detect the HTE-bound LigB proteins. The similar procedure was utilized to fine-map the HTE-binding sites on LigB12 and LigB7. A total of 12 different tag-free LigB5/LigB12 and LigB7/LigB10 chimeras plus wild-type LigB5, LigB12, LigB7, and LigB10 (control) were coated on the wells. For pinpointing the key HTE-binding residues on LigB12, five tag-free LigB12 mutants (F1054A, D1061N, A1065K, D1066A, and E1088A) and wild-type LigB12 were individually immobilized on the wells. Then, five micromolars of histidine tagged HTE17-20, HTE20, and HTE20N were applied to either Lig protein chimeras or LigB12 mutants coated wells. The binding of truncated HTE to chimeric Lig proteins or various LigB12 mutants were detected by anti-His antibodies. Finally, 100 μL of TMB substrates were added and the microtiter plates were read at 630 nm by an ELISA plate reader (Biotek EL-312, Winooski, VT). Each OD_630_ value shown in the figures represents the mean of three independently determinants ± 1 standard deviation from three replicates. The equilibrium dissociation constant (*K*_*D*_) was calculated by fitting the data to the equation indicated below:

$$\begin{array}{l} \text{O}{\text{D}}_{630}=\frac{\text{O}{\text{D}}_{630\text{max}}\left[\text{Lig proteins}\right]}{K_{D}+\left[\text{Lig proteins}\right]} \end{array}$$

### Binding kinetics study by surface plasmon resonance (SPR)

To investigate the binding kinetics between Lig proteins and HTE truncations, SPR was performed by using a Biacore 3000 instrument (GE Healthcare). In brief, 50 μg.ml^−1^ tag free LigB12 in 10 mM acetate buffer (pH 4.0) were, respectively immobilized on different flow cells of a CM5 sensor chip until reaching a level of 1,000 resonance units. The control flow cell was activated and blocked by the same reagents (NHS-EDC and ethanolamine) used for LigB-coated cells except that no protein was added for the control cell. Serial concentrations of HTE truncates (0, 0.3125, 0.625, 1.25, 2.5, and 5 μM of HTE17-20, HTE19-20, HTE20, HTE20N, and HTE20C) were individually injected to the flow cells in PBS buffer at a flow rate of 30 μL/min. The chip surface was regenerated by removal of analyte with a regeneration buffer (10 mM glycine-HCl at pH 3.0). All sensogram data were recorded at 25°C and normalized by subtracting the data from the control flow cell. To determine the kinetics parameters (*k*_*on*_ and *k*_*off*_) and the binding affinity (*K*_*D*_) of LigB-HTE interactions, the binding sensograms were fitted by BIAevaluation software using one-step biomolecular association reaction model (1:1 Langmuir model) version 3.0 model, which gave the optimal mathematical fits with the lowest χ-values.

### Binding experiments by fluorescence spectroscopy

Given that single Ig-like domain of LigB contains only one tryptophan and that HTE truncates do not contain any tryptophan, the binding of HTE to LigB proteins could be examined by steady state fluorescence spectrocopy using Hitachi F4500 spectrofluorometer (Hitachi. San Jose, CA). The intrinsic tryptophan fluorescence of LigB proteins (2 μM) alone was monitored at 25°C by exciting the solutions at 295 nm and measuring the emission in the 305–400-nm regions. In the same spectrum mode, a series of 2-fold dilutions of HTE20 or HTE20N (10 to 0.625 μM) in PBS buffer were gradually titrated into LigB solutions, and the fluorescence intensity was individually recorded after 5 min of incubation. The fluorescence intensity of each HTE20 or HTE20N without Lig proteins was also recorded and used to subtract the spectra from the corresponding HTE20 or HTE20N with Lig proteins. To calculate the dissociation constant (*K*_*D*_), the changes of fluorescence intensity of various concentrations of LigB-HTE mixtures were measured at 315 nm and fitted with equation shown below using OriginLab software (version 7.0),

$$\begin{array}{l} F_{\text{max}}-F=\frac{\left(F_{\text{max}}-F_{\text{min}}\right)\left[\text{tropoelastin}\right]}{K_{D}+\left[\text{tropoelastin}\right]} \end{array}$$

where *F*_max_ is the fluorescence intensity of Lig proteins in the absence of HTE; *F*_min_ indicates the fluorescence intensities of Lig proteins saturated with HTE. In addition, *F* is the fluorescence intensities of Lig proteins in the presence of various concentrations of HTE. All of the measurements were corrected for dilution and for inner filter effect.

### Adhesion assay

To investigate if the recombinant HTE truncations can block the binding of LigB4 or LigB12 to tropoelastin-producing WI-38 cells, competitive ELISA binding assays were conducted. The WI-38 cells were seeded at the concentration of 10^5^ cells per well in a 96-well-tissue culture plate and incubated at 37°C overnight. After the cell monolayer developed, the culture supernatant was replaced with EMEM containing 10% FBS with no antibiotics for 1 h. Five micromolars of GST-LigB4 or GST-LigB12 was pre-incubated with serial dilutions of HTE17-20, HTE20N, and HTE20C (10 to 0.156 μM) for 1 h prior to the addition to WI-38 cells for additional 3 h incubation at 37°C. The unbound proteins were removed from cell surface by washing the plates with 0.05% PBS-T for three times. Subsequently, HRP-conjugated rabbit anti-GST antibodies (1:1,000) in PBS containing 1% BSA were used to detect the cell surface associated Lig proteins. Finally, after three times washing, TMB substrates were added as previously described. For the bacterial adhesion assay, we initially tested if LigA-expressing *L. biflexa* (Patoc ligA) could adhere to cell surface of WI-38 cells. A serial 2-fold dilution of spirochetes was added to overnight-grown WI-38 cells in microtiter plates for 3 h at 37°C. The unbound bacteria were removed by three times washes with 0.05% PBS-T, while bound bacteria were fixed with 4% paraformaldehyde for 30 min. Fixatives were quenched by 125 mM of glycine and the quenching reagents were rinsed off with PBS. Lastly, hamster anti- *L. biflexa* polyclonal antibodies (1:1,000) and goat anti-hamster IgG antibodies conjugated with HRP (1:2,000) were used as primary and secondary antibodies to detect WI-38 cells-bound spirochetes. To examine if the HTE truncations can decrease *L. biflexa* (Patoc *ligA*) binding to WI-38 cells, a competitive bacterial adhesion assay was performed. Briefly, 10^7^ cells ml^−1^ of Patoc *ligA* were pre-incubated with various concentrations of HTE17-20, HTE20N, and HTE20C (10 to 0.156 μM) for 1 h before being added to overnight-grown WI-38 cells (10^5^ per well) at 37°C. Following 3 h incubation with WI-38 cells and then three times washes with 0.05% PBS-T, the cell-bound spirochetes were fixed with 4% paraformaldehyde and quenched by 125 mM of glycine as preciously described. Similarly, hamster anti- *L. biflexa* polyclonal antibodies and HRP conjugated goat anti-hamster IgG antibodies were used to measure the cell binding levels of spirochetes.

### Statistical analysis

GraphPad Prism 6.0 (GraphPad Software, Inc.), ANOVA tests, and *t*-tests were used to analyze the data. The *p* < 0.05 is considered as statistically significant, while the *p* > 0.1 is considered as statistically insignificant.

## Results

### The proline-rich hydrophobic domains of tropoelastin are the binding sites for LigB4 and LigB12

**Figure 2:**
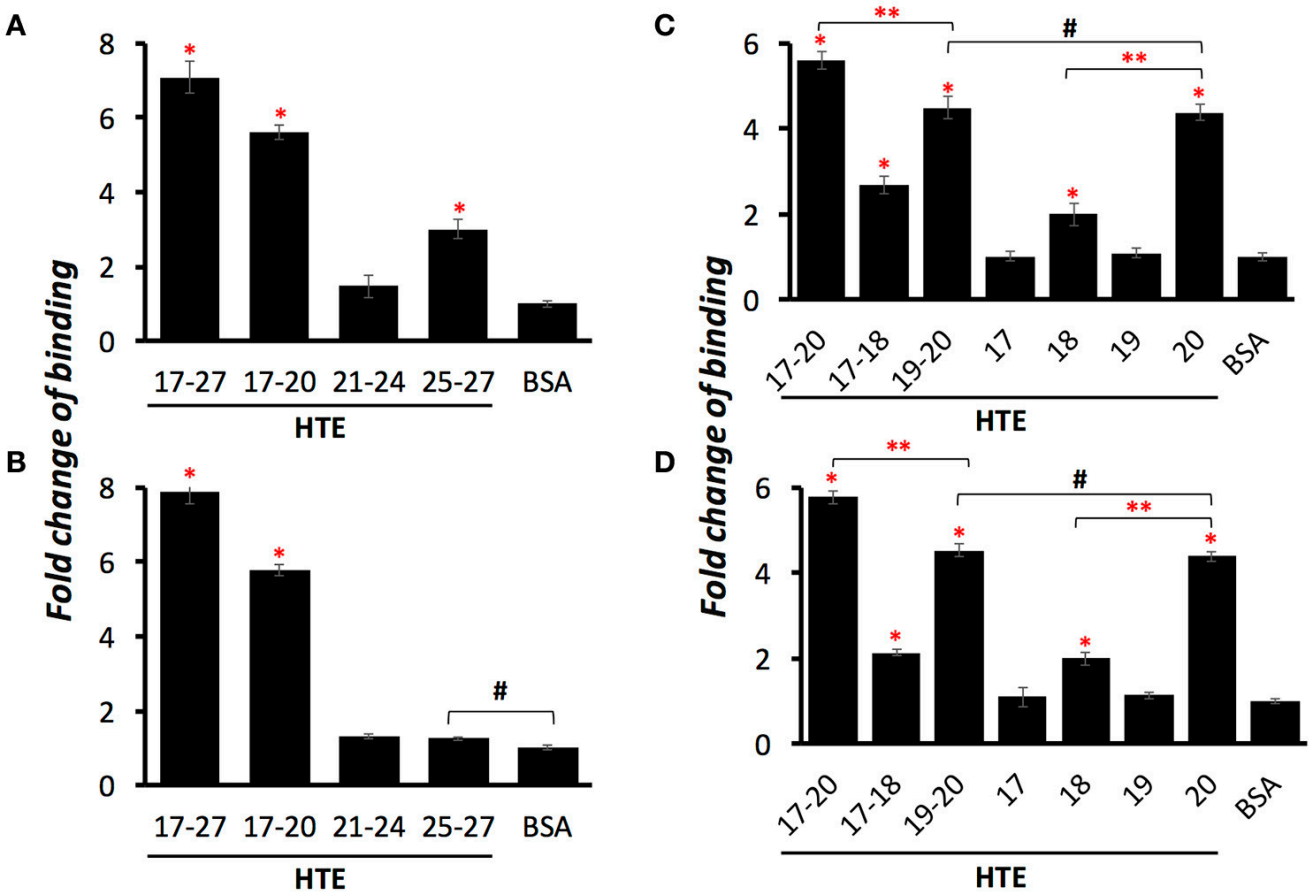
**LigB Ig-like domains bind to proline-rich hydrophobic domains of HTE**. A series of HTE truncates (1 μM/well) or BSA (negative control) were immobilized on the microtiter plates, and then 5 μM of GST tagged LigB12 **(A,C**) or LigB4 **(B,D**) was individually applied to HTE-or BSA-coated wells. The binding of GST-tagged proteins to different HTE fragments were detected by ELISA using HRP-conjugated anti-GST antibodies. Differences of binding are shown as fold change compared to BSA. All experiments were conducted three times independently and the results illustrated as the mean ± 1 standard deviation. Asterisks indicate that binding of GST-tagged LigB to HTE was significantly greater than that to BSA (*p* < 0.05, *t*-test). Double asterisks indicate a significant difference of binding between two groups (*p* < 0.05, *t*-test). Pound signs indicate that the difference of binding between these two groups is statistically insignificant (*p* > 0.1, *t*-test).

Previously, our group had identified that LigB Ig-like domains bound to 17th to 27th exon of human tropoelastin (HTE17-27). Based on the binding affinity to HTE, the repeated domains could be classified into three groups: strong binders (LigB4 and LigB12), moderate binders (LigB7′-8 and LigB9), and weak binders (the rest of LigB repeats; Lin et al., 2009). To further pinpoint the minimal binding site for LigB, HTE17-27 was truncated into three fragments: HTE17-20, HTE21-24, and HTE25-27 as indicated in Figure 1. The two strongest HTE-binding partners, LigB4 and LigB12, were added to microtiter wells coated with different HTE truncates including HTE17-27 (positive control) and BSA (negative control). As expected, both LigB4 and LigB12 bound strongly to HTE17-27, while none of them interacted with BSA (Figures 2A,B). Among all HTE truncates, HTE17-20 was consistently recognized by both LigB12 and LigB4. This strong interaction between HTE17-20 and Lig proteins was 5.7-fold greater than negative control (*p* < 0.05). HTE25-27 also showed comparable binding to LigB12 (3-fold higher than negative control, *p* < 0.05), but it did not interact with LigB4 at significant levels. In addition, there was no significant binding of HTE21-24 to either LigB4 or LigB12.

HTE17-20 is composed of alternating cross-linking domains (HTE17, HTE19) and proline-rich hydrophobic domains (HTE18, HTE20). To investigate whether the ability of LigB4 or LigB12 binding to different domains of HTE17-20 is distinct, the affinity of LigB proteins to respective single domain (HTE17, HTE18, HTE19, and HTE20) or double domain (HTE17-18 and HTE19-20) was assessed by ELISA. As shown in Figures 2C,D, HTE19-20 was strongly recognized by both LigB12 and LigB4, the binding of which were 4.5-fold higher than negative control (*p* < 0.05). This interaction between HTE19-20 and LigB4 or LigB12 was slightly weaker than that between HTE17-20 and LigB proteins (*p* < 0.05). On the other hand, HTE17-18 was recognized by LigB12 and LigB4 to a lesser extent, the interactions of which were 2.1–2.7-fold higher than negative control (*p* < 0.05). For the single domain HTE truncations, HTE20 displayed the strongest binding to LigB12 and LigB4 (4.4-fold higher than negative control), while the binding affinity was not significantly different from the interaction between HTE19-20 and LigB proteins (*p* > 0.1). This could explain the fact that HTE19 exhibited no binding affinity to both LigB12 and LigB4. Additionally, HTE18 showed distinguishable binding to either LigB12 or LigB4 (2-fold higher than negative control, *p* < 0.05), but this interaction was much weaker than HTE20-LigB interaction (*p* < 0.05). Taken together, these findings demonstrate that both LigB12 and LigB4 recognized HTE17-20, HTE19-20 and more specifically HTE20, suggesting that proline-rich hydrophobic domains of HTE are the binding sites for LigB Ig-like domains.

### LigB12 binds to HTE17-20, HTE19-20, and HTE20 with submicromolar affinities

To precisely characterize the real-time binding kinetics of LigB-HTE interactions, the aforementioned HTE truncates (HTE17-20, HTE19-20, and HTE20) were used to analyze the interaction with LigB12 by surface plasmon resonance (SPR). By flowing the individual HTE fragments through a LigB12-coated CM5 sensor chip, association rate constants (*k*_*on*_), dissociation rate constants (*k*_*off*_) and equilibrium dissociation constant (*K*_*D*_) were obtained. As shown in Table 1 and Supplementary Figure 1A, HTE17-20 exhibited a strong binding to LigB12 with submicromolar affinity (*k*_*on*_ = 1.3 × 10^3^ ± 0.3 M^−1^ s^−1^, *k*_*off*_ = 1.1 × 10^−3^ ± 0.7 s^−1^, *K*_*D*_ = 0.85 ± 0.04 μM). HTE19-20 (Supplementary Figure 1B) and HTE20 (Supplementary Figure 1C) also displayed comparable binding affinities to LigB12 with kinetic parameters *k*_*on*_ = 3.1 × 10^3^ ± 0.7 M^−1^ s^−1^, *k*_*off*_ = 4.3 × 10^−3^ ± 0.5 s^−1^, *K*_*D*_ = 1.34 ± 0.06 μM for HTE19-20 and *k*_*on*_ = 3.4 × 10^3^ ± 0.7 M^−1^ s^−1^, *k*_*off*_ = 3.2 × 10^−3^ ± 0.3 s^−1^, *K*_*D*_ = 0.94 ± 0.04 μM for HTE20. In comparison with the affinity of HTE17-20 to LigB12, the smaller constructs (HTE19-20 and HTE20) had slightly lower affinities to Lig proteins and presented a fast association and fast dissociation kinetics. The binding affinity of HTE20 to LigB12 was also obtained by calculating the change of tryptophan fluorescence intensity as increasing concentrations of HTE20 was titrated into LigB12 (Table 1 and Supplementary Figure 1D). In agreement with the SPR data, the measured *K*_*D*_ was equal to 0.97 ± 0.03 μM. To sum up, HTE20, a single proline-rich hydrophobic domain, retained the sub-micromolar affinity to LigB12.

**Table 1:** **Dissociation constants and kinetic data for different HTE truncations interacting with LigB12, as determined by surface plasmon resonance and fluorescence spectroscopy**.

**Analytes**	**Surface plasmon resonance**			**Fluorescence spectroscopy**
	***K_D_* (μM)**	***k_on_*(M^−1^ s^−1^)**	***k_off_* (s^−1^)**	***K_D_* (μM)**
HTE17-20	0.85 ± 0.04	1.3 × 10^3^ ± 0.3	1.1 × 10^−3^ ± 0.7	n.d.^a^
HTE19-20	1.34 ± 0.06	3.1 × 10^3^ ± 0.7	4.3 × 10^−3^ ± 0.5	n.d.^a^
HTE20	0.94 ± 0.04	3.4 × 10^3^ ± 0.7	3.2 × 10^−3^ ± 0.3	0.97 ± 0.03
HTE20N	0.78 ± 0.05	2.7 × 10^3^ ± 0.4	2.1 × 10^−3^ ± 0.2	0.82 ± 0.06

*All values represent the mean ± 1 standard deviation from three independent experiments*.

a *n.d., Not determined*.

### Amino terminal region of HTE20 (HTE20N) is the minimal binding site for LigB4 and LigB12

**Figure 3:**
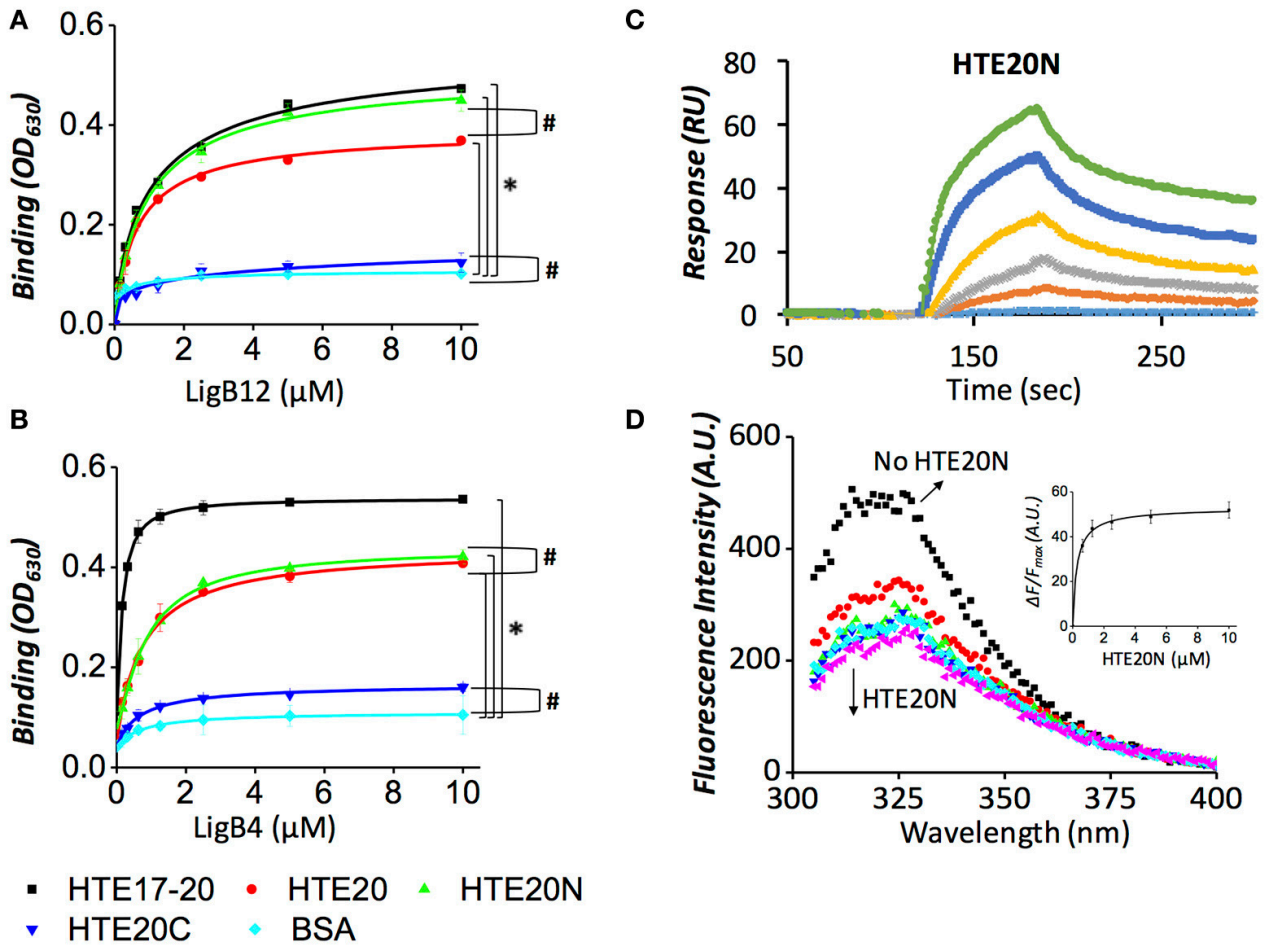
**Both LigB4 and LigB12 specifically bind to the N-terminal region of HTE20 (HTE20N). (A,B)**. ELISA was performed to examine the binding affinities of HTE20 truncates to LigB12 **(A)** or LigB4 **(B)**. Various concentrations of GST tagged LigB proteins (0, 0.16, 0.32, 0.63, 1.25, 2.5, 5, and 10 μM) were applied to HTE17-20 (positive control), HTE20, HTE20N, HTE20C, and BSA (negative control) coated wells, respectively (1 μM/well). The binding of LigB to HTE truncates was evaluated by ELISA. Asterisks indicate that binding of LigB to HTE truncates was significantly greater than that to BSA (*p* < 0.05, *t*-test). Pound signs indicate that the difference of binding between the two groups is statistically insignificant (*p* > 0.1, *t*-test). **(C)**. SPR analysis of LigB12-HTE20N interaction was conducted by flowing the HTE20N (5 to 0.31 μM, 2-fold serial dilution) through LigB12-coated CM5 sensor chip. The sensogram shown was a representative of three independent experiments. The *K*_*D*_, *k*_*on*_, and *k*_*off*_-values of this interaction are shown in Table 1 and were obtained from the average of these three experiments. **(D)**. Intrinsic fluorescence spectrum of LigB12 in the presence and absence of HTE20N. Different concentrations (10 to 0.625 μM) of HTE20N were titrated into LigB12 (2 μM), and the extent of the fluorescence quenching of LigB12 was monitored. As inset, the changes of fluorescence intensity of various concentrations of LigB-HTE mixtures were measured at 315 nm and were plotted as function of HTE20N concentrations. The saturation curve was then fitted with the equation stated in Section Materials and Methods to calculate the dissociation constant (*K*_*D*_), which is shown in Table 1. Shown was a representative of six experiments performed on three separate occasions.

To identify the minimal binding sites for LigB proteins, HTE20 was further truncated into two fragments, the N-terminal part (HTE20N) containing unique basic and aromatic amino acids and the C-terminal part (HTE20C) consisting of classic VPGVG repeats. The binding of each truncated HTE to LigB12 or LigB4 was analyzed by ELISA. HTE17-20, HTE20, and BSA were also included as positive and negative control. As expected, HTE17-20 showed the greatest binding affinity to either LigB12 or LigB4 (*K*_*D*_ = 0.78 μM), which is significantly greater than negative control (*p* < 0.05; Figures 3A,B). Interestingly, HTE20N exhibited a significant binding to both Lig proteins (*K*_*D*_ = 0.86 μM; *p* < 0.05, compared to negative control). These HTE20N-LigB interactions were at statistically similar levels to HTE20 binding to Lig proteins (*p* > 0.1), which suggests HTE20N maintained a complete binding site for LigB12 and LigB4. In addition, HTE20C totally lost the capacity for recognizing Lig proteins (*p* > 0.1, compared to negative control). To gain more insight into the interaction of Lig proteins with HTE20N in label-free and liquid phase settings, LigB12 was chosen for characterization by SPR and steady state fluorescence spectroscopy. As shown in Figure 3C, HTE20N displayed a tight association with LigB12 as measured *K*_*D*_ at 0.78 ± 0.05 μM, which is comparable to the binding affinity of HTE17-20 to LigB12. Rate constants *k*_*on*_ (2.7 × 10^3^ ± 0.4 M^−1^ s^−1^) and *k*_*off*_ (2.1 × 10^−3^ ± 0.2 s^−1^) of HTE20N-LigB12 interaction were also similar to HTE17-20-LigB12 interaction. Consistent with SPR data, the binding affinity (*K*_*D*_) of HTE20N to LigB12 calculated from steady state fluorescence spectroscopy was 0.82 ± 0.06 μM (Figure 3D). LigB12 only contains one tryptophan buried in the hydrophobic core. The continuous quenching of tryptophan fluorescence intensity due to gradually increased ratio of HTE20N to LigB12 suggests that the HTE binding sites might be in the proximity of core tryptophan. In summary, we pin down the minimal binding site, HTE20N, essential for binding to LigB with strong affinity.

### Basic and aromatic amino acids are the key residues for HTE20N recognized by LigB

**Figure 4:**
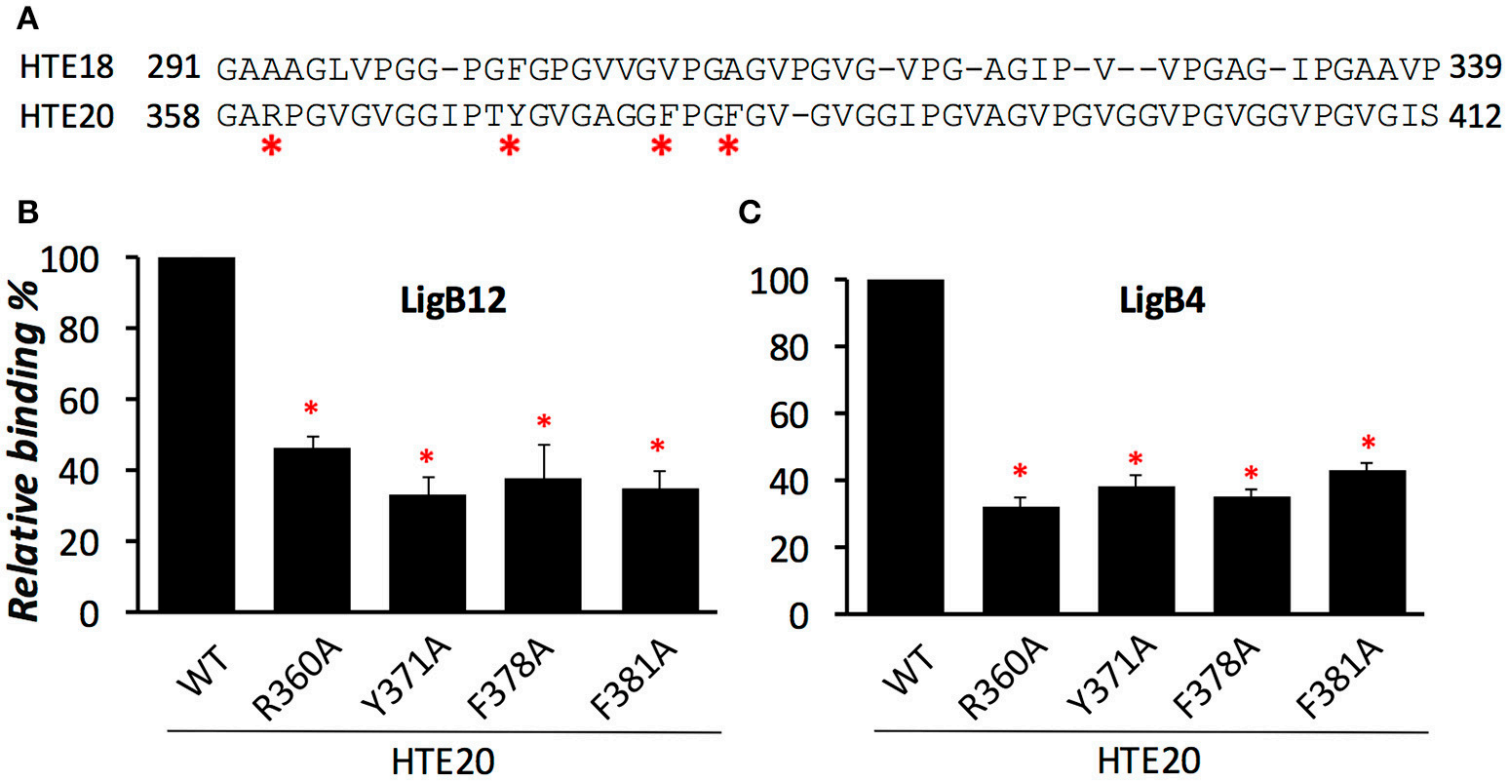
**Basic residue R360 and aromatic residues Y371, F378, and F381 from HTE20 are the critical residues for association with LigB proteins. (A)**. Sequence alignment of HTE18 and HTE20 was created by ClustalW and the non-conserved residues were indicated by asterisks. **(B,C)**. The binding of wild type or four HTE20 point mutants to LigB12 **(B)** and LigB4 **(C)** were assessed by ELISA. Five micromolars of GST tagged LigB were added to WT or HTE20 mutants coated wells (1 μM/well). Binding is expressed relative to binding by wild type HTE20. Each bar represents the mean of three independent determinations ± 1 standard deviation. Asterisks indicate that binding was significantly different from binding of wild type HTE to the respective LigB protein (*p* < 0.05, *t*-test).

Although both HTE18 and HTE20 could be recognized by LigB, the binding affinity of HTE20 to LigB is two times greater than HTE18 to LigB (*p* < 0.05, Figure 2). To investigate the distinct LigB binding abilities of these two Pro-rich hydrophobic domains, the amino acid sequences of HTE18 and HTE20 were aligned by ClustalW. As indicated in Figure 4A, HTE20 has an arginine at the 3rd position, while HTE18 has an alanine at the corresponding site. Other bulky aromatic acids such as Y371, F378, and F381 also specifically present in HTE20 instead of HTE18. Given that the differences of these characteristic amino acids in HTE20, we hypothesize that they might be involved in the interaction with LigB. To this end, we generated four HTE20 mutants R360A, Y371A, F378A, and F381A and examined the abilities of these mutants binding to LigB12 and LigB4. Wild-type HTE20 (WT) was included in the binding assay, and the relative binding (%) of each mutant was calculated in relation to WT. As shown in Figure 4B, all HTE20 mutants partially lost the capacities for interacting with LigB12. Mutants Y371A and F381A, only retained 35% of WT binding affinity to LigB12, while F378A and R360A decreased the binding by 62 and 54% as opposed to WT. Likewise, the interaction between LigB4 and each HTE20 mutant was decreased (Figure 4C). Two mutants R360A and F378A only had 32 and 35% of original binding affinity to LigB4. The binding of Y371A and F381A to LigB4 was also decreased by 61 and 54% compared to WT. Nevertheless, the binding level of each HTE20 mutant to LigB12 or LigB4 was not significantly different from each other. Overall, these results suggest that positive charged and aromatic amino acids on HTE20 play a role in binding to LigB12 and LigB4.

### HTE17-20, HTE20, and HTE20N bind to the first β-sheet of LigB12

**Figure 5:**
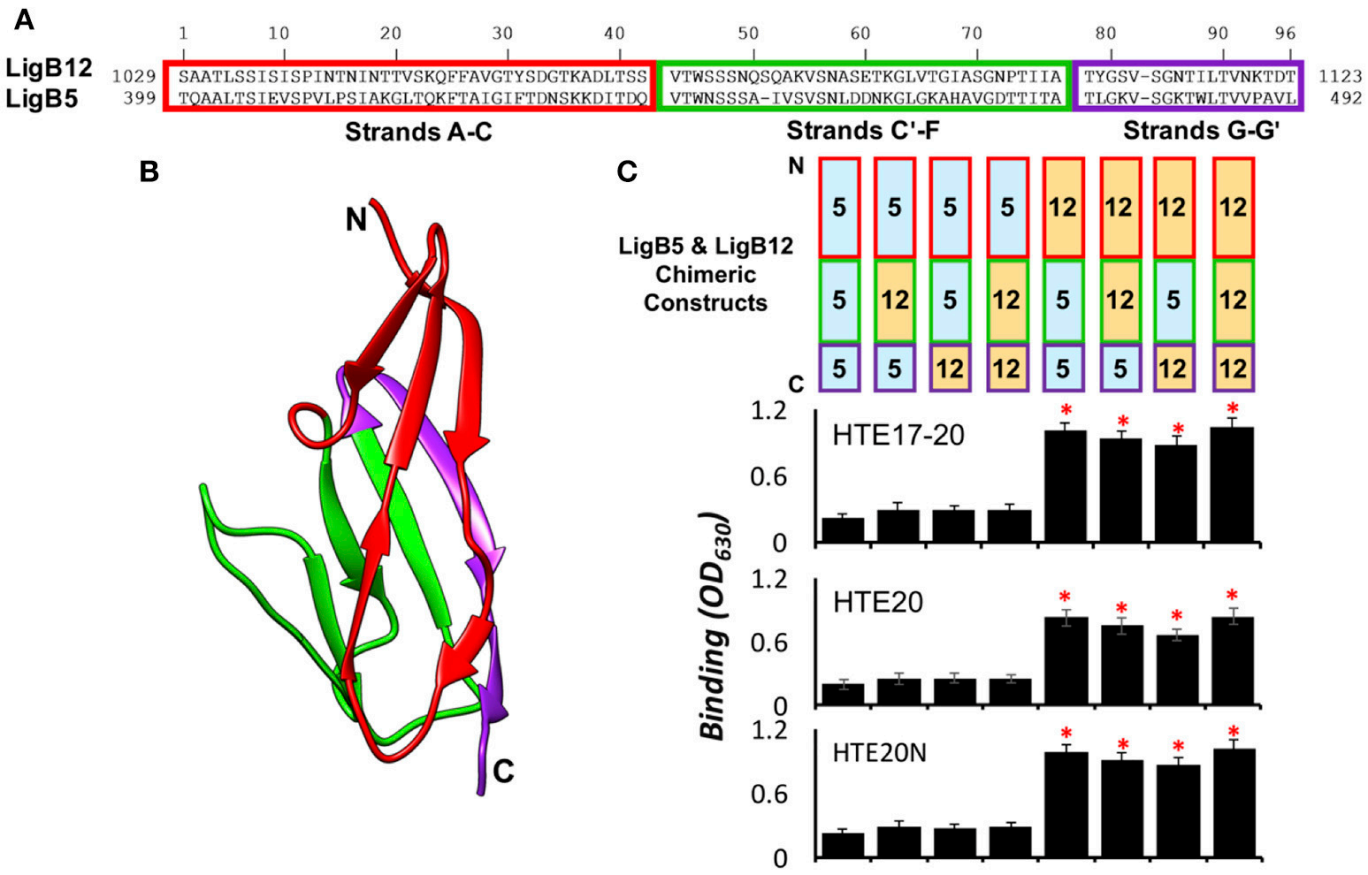
**HTE17-20, HTE20, and HTE20N bind to the first β-sheet of LigB12. (A)**. Amino acids sequence alignment of LigB12 and LigB5. The regions of three subdomain sequences (**A–C,C'–F,G–G'**) were enclosed in red, green and purple rectangles. **(B)**. High resolution structure of LigB12 showing three distinct solvent-exposed surfaces, composed of strands A–C (shown in red), strands C'–F (shown in green) and strands G–G' (shown in purple). N-terminal end and C-terminal end of LigB12 structure was also indicated as N and C. **(C)**. (Top panel) Schematic representation of the chimeric LigB5/LigB12 constructs used in this study. (Bottom panel) The binding of LigB5/LigB12 chimeras to HTE17-20 (top), HTE20 (middle), or HTE20N (bottom) was analyzed by ELISA. Sole LigB5 was also included as a negative control (Lin et al., 2009). Five micromolars of histidine tagged HTE17-20, HTE20, and HTE20N were applied to LigB5/LigB12 chimeras coated wells (1 μM/well). The binding of truncated HTE to Lig proteins was detected by anti-His antibodies. All experiments were conducted in triplicates, each bar represents the mean of three independent determinations ± 1 standard deviation. Asterisks indicate that binding was different from binding of HTE to the LigB5 (*p* < 0.05, *t*-test).

The HTE binding sites within individual Ig-like domains was further investigated by designing chimeras of two equal-length domains, LigB5 and LigB12. Given that only LigB12 can be recognized by HTE, the region of LigB12 swapped with inert LigB5 counterpart can be used to identify its binding ability to HTE (Lin et al., 2009). Based on the homologous LigB12 structure (Ptak et al., 2014), two chimeric swapping points were used to establish three distinct regions: (1) β-strands A–C, (2) β-strands C'–F, and (3) β-strands G–G' (Figures 5A,B). The first two regions of the chimeras were separated at the half helix break in β-strand C, effectively dividing the top and bottom halves of the Ig-like domain β-sandwich. The third chimera region was included to determine if an important surface is formed on the non-covalent edge of the β-sandwich. Including the wild-type LigB5 and LigB12 domains, eight possible combinations of the three regions could be generated on single Ig-like domains (Figure 5C). HTE17-20, HTE20, and HTE20N were subjected to an ELISA binding screen against the eight LigB5/LigB12 recombinant proteins. As expected, none of HTE truncates bound to wild-type LigB5, while all truncates strongly recognized wild-type LigB12. As for the chimeric proteins, all HTE truncates showed binding to only three of the six chimeras with a clear region-specific pattern (Figure 5C). Among these chimeras, LigB12-5-5 maintained the full binding affinity to either HTE17-20 HTE20, or HTE20N, which has no significant difference from the binding of wild-type LigB12 to either HTE truncates (*p* > 0.1). LigB12-12-5 and LigB12-5-12 also displayed great binding ability by retaining nearly 90 and 80%, respectively, of wild-type LigB12 binding capacity to both HTE truncates. On the other hand, the other three chimeras, LigB5-5-12, LigB5-12-12, and LigB5-12-5 completely lost the ability to interact with HTE17-20, HTE20, or HTE20N (in relation to negative binding control, *p* > 0.1). It has been shown that LigB7 can bind to HTE although the affinity is weaker than that of LigB12 binding to HTE (Lin et al., 2009). Intriguingly, all HTE truncates preferentially bound to wild-type LigB7, LigB7-7-10, and LigB7-10-10 as well as LigB7-10-7 (Supplementary Figures 2A–C) but not to other LigB7/10 chimeras. To be noted, all LigB chimeras used here maintained the structural integrity as their parental wild-type LigB (examined by circular dichroism, CD, data not shown.) All of these findings indicate the binding site for tropoelastin seems to locate at the first β-sheet (β-strands A–C) of LigB Ig-like domains.

### Phe1054, Asp1061, Ala1065, and Asp1066 are pivotal for the association of LigB12 and HTE

**Figure 6:**
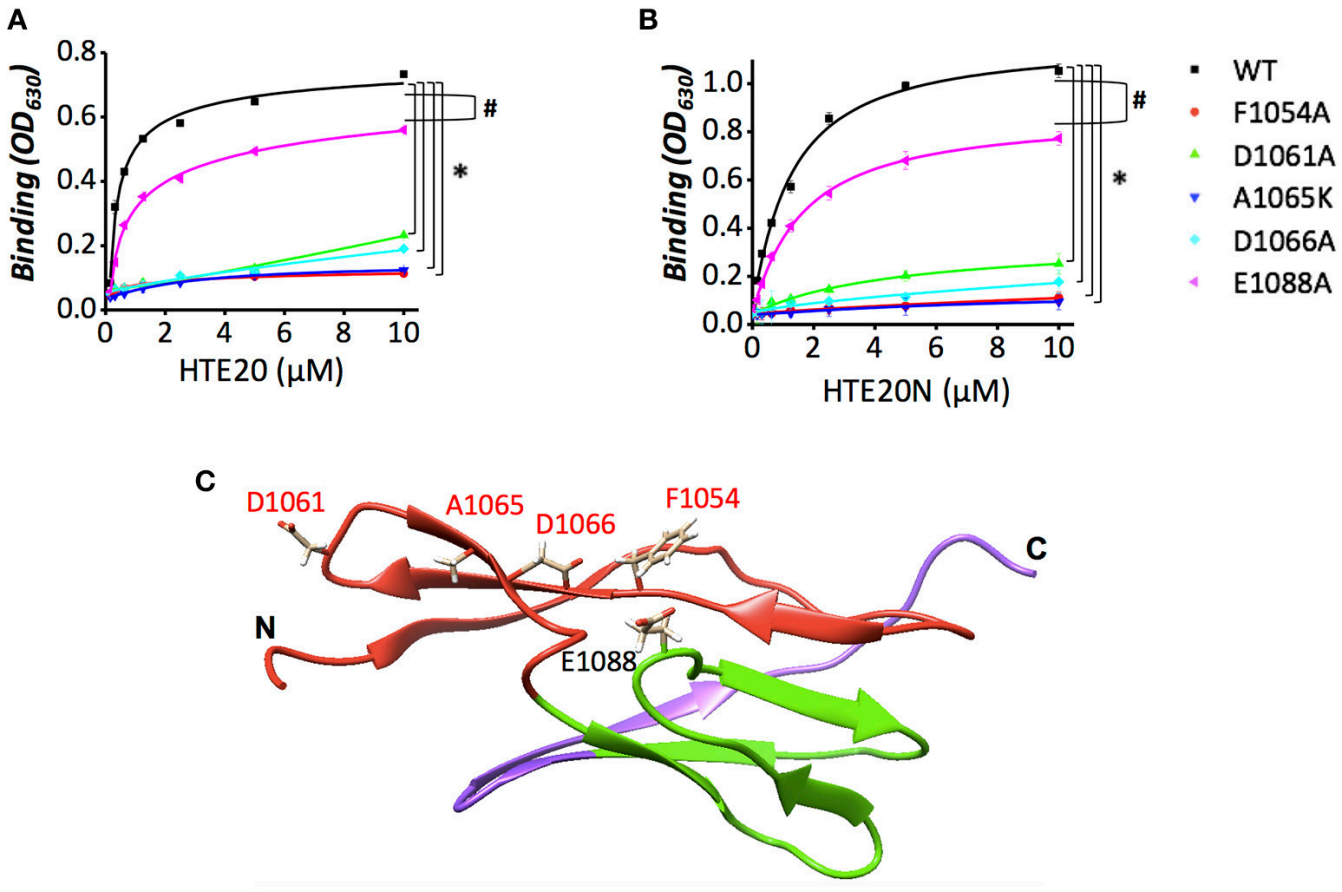
**Replacement mutations of F1054, D1061, A1065, or D1066 reduce the binding activity of LigB12 to HTE. (A,B)** The binding activity of LigB12 wild-type (WT) and four mutants located on a potential HTE-binding site of LigB (D1061N, D1066A, A1065K, and F1054A) to HTE20 was measured by ELISA. LigB-E1088A was also included as a negative control. His tagged HTE20 or HTE20N (0, 0.16, 0.32, 0.63, 1.25, 2.5, 5, and 10 μM) were applied to LigB12 (positive control), LigB12 point mutants (F1054A, D1061N, A1065K, D1066A, and E1088A), or BSA (negative control, data not shown) coated wells (1 μM/well). The binding of HTE20 or HTE20N to different LigB12 constructs was detected by anti-His antibodies. All experiments were conducted in three independent trials and the mean ± 1 standard deviation of the results was shown. Asterisks indicate that binding of LigB12 mutants to HTE truncates was significantly lower than LigB12 WT (*p* < 0.05, *t*-test). Pound signs indicate that binding of E1088A to HTE truncates was statistically similar to LigB12 WT (*p* > 0.1, *t*-test). **(C)**. The potential HTE-binding surface on the structure of LigB12. Four critical residues (D1061, A1065, D1066, and F1054) of LigB12 involved in a potential HTE-binding site were highlighted in red. E1088, which is not located on the potential HTE-binding site was shown in black.

Given that positively charged and aromatic amino acids of HTE20 might contribute to the HTE-Lig interaction, and that HTE specifically bound to the first β-sheet of LigB12, we hypothesized that the negatively charged and aromatic amino acids on the first β-sheet of LigB12 might be critical for HTE20 binding. To this end, a series of LigB12 mutants F1054A, D1061A, and D1066A were generated and the binding ability of these mutants to HTE17-20 (data not shown), HTE20 and HTE20N were examined by ELISA. In addition, E1088, an acidic residue from second β-sheet of LigB12, was chosen as internal control. As shown in Figures 6A,B, E1088A displayed a comparable strong binding to either HTE20 or HTE20N, the binding affinity (ELISA, *K*_*D*_ = 0.87 μM) of which was no difference from that of wild-type LigB12 to HTE (*p* > 0.1). Intriguingly, F1054A, D1061A, and D1066A all lost the capacities to interact with either HTE truncate (*p* < 0.05, compared to wild-type LigB12), presenting only 15, 32, and 26% of wild-type LigB12 binding capacities. Partial sequence alignment of LigB Ig-like domains revealed that unique small amino acids (e.g., Ala or Ser) are specifically present at HTE strong binders (position 615 of LigB7 and position 1,064 of LigB12), while large polar residues (e.g., Lys or Gln) are located at the corresponding position of LigB5, LigB10, and other weak binders (Supplementary Figure 2D). Thus, we generated A1065K and tested its binding ability to HTE truncates. As a result, this LigB12 mutant largely diminished the binding to HTE20 (Figure 6A) and HTE20N (Figure 6B; *p* < 0.05, compared to wild-type LigB12). The chromatographically purified LigB12 mutants and wild-type were 95% pure (Supplementary Figure 3). The secondary structures of LigB12 mutants have also been examined by CD and did not show any significant change compared to the wild-type (Hsieh et al., 2016). In conclusion, these results demonstrate that F1054, D1061, A1065, and D1066 from LigB12 play a major role in the association with HTE. The potential HTE-binding interface on LigB12 are also shown as Figure 6C.

### HTE17-20 and HTE20N abolish the binding of LigB Ig-like repeats to human lung cells

**Figure 7:**
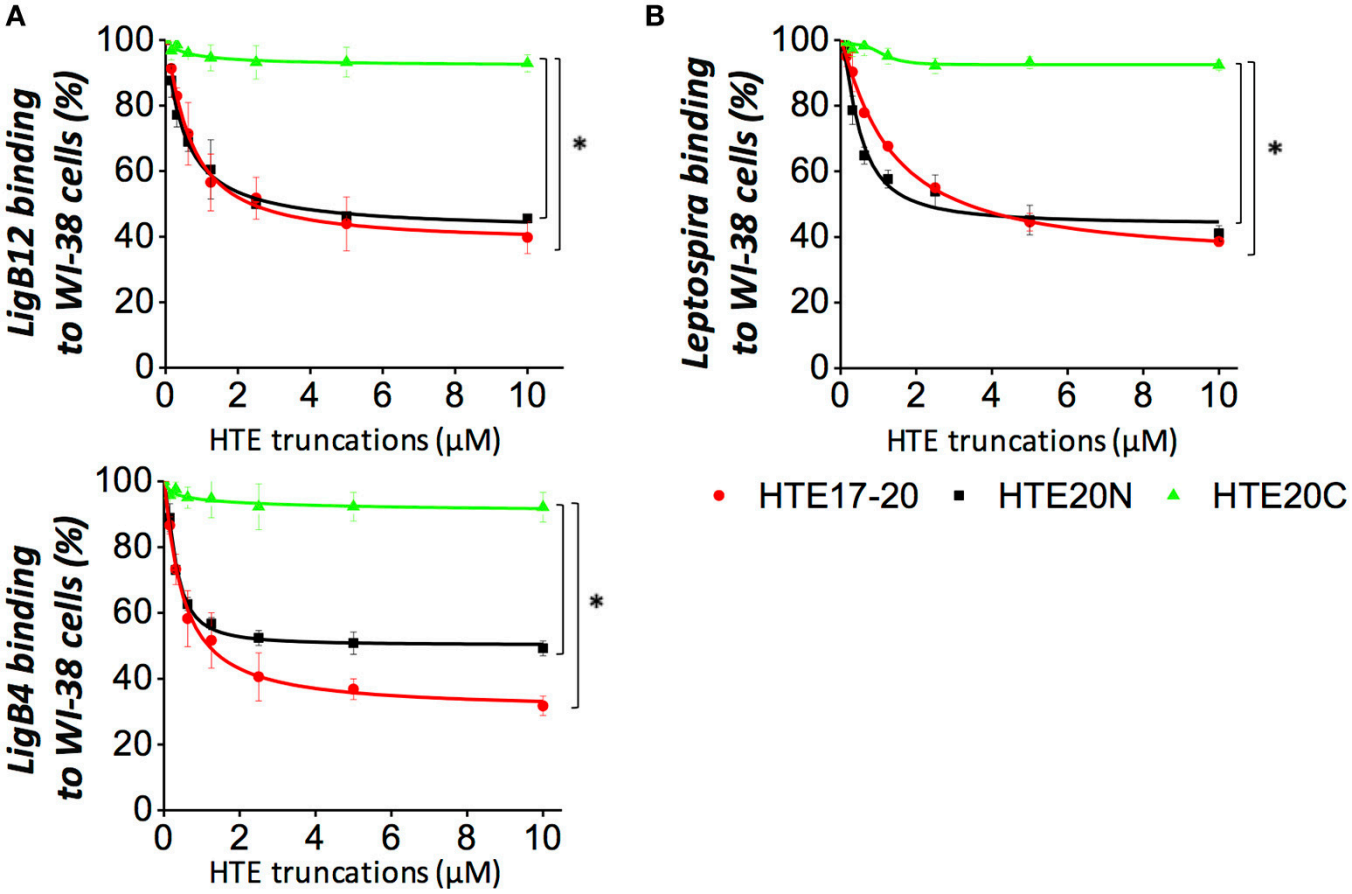
**HTE17-20 and HTE20N could partially inhibit the leptospiral adhesion to human embryonic lung cells. (A)** The binding of LigB12 (top panel) or LigB4 (botton panel) to human embryonic lung fibroblasts WI-38 cells in the presence or absence of exogeneous HTE truncates. Five micromolar of GST tagged LigB4 or LigB12 was treated with serial dilutions of HTE17-20, HTE20N, or HTE20C (10 to 0.156 μM) prior to be incubated with WI-38 cells. After 1 h of incubation, the cell-binding activity of LigB was evaluated by ELISA. **(B)** A high passage, non-infectious *L. biflexa* strain Patoc expressing LigA was treated with a serial dilution of HTE17-20, HTE20N, or HTE20C (10 to 0.156 μM) prior to be incubated with WI-38 cells. After 3 h of incubation, the binding of spirochetes to lung cells was assessed by ELISA using hamster antisera against *L. biflexa*. All experiments were conducted in three independent trials and the results were illustrated as the mean ± 1 standard deviation. Asterisks indicate that the inhibition caused by HTE17-20 or HTE20N was significant higher than that caused by HTE20C (*p* < 0.05, *t*-test).

Our group has shown that Lig proteins mediate the leptospiral attachment to host cells by interacting with fibronectin (Lin et al., 2010). To examine whether LigB Ig-like repeats could adhere to human cells through binding to tropoelastin, HTE-producing human embryonic lung fibroblasts (WI-38) were used for the adhesion assays. GST-tagged LigB4 or LigB12 was individually applied to WI-38 cell monolayer and the binding level was detected by anti-GST antibodies. As expected, GST protein alone did not show any detectable binding to WI-38 cells, while either GST-LigB4 or GST-LigB12 exhibited a dose-dependent association to the cells (data not shown). Provided that the HTE-LigB interaction presented submicromolar binding affinity, we further investigated if this strong interaction could block the binding of LigB to human cells. Either GST-LigB4 or GST-LigB12 were pre-treated with various concentrations of recombinant HTE17-20 or HTE20N prior to the addition to WI-38 cells. HTE20C treated LigB proteins were also included in the study as a negative control. As shown in Figure 7A, molar excessive amounts of HTE20C had no significant effect on the LigB adhesion to WI-38 cells. However, both HTE17-20 and HTE20N could partially abolish the LigB binding to the cells. This abolishment was significant compared to that of HTE20C treated group (*p* < 0.05). The highest concentration of HTE17-20 could decrease 68% of LigB4 and 60% of LigB12 binding to WI-38 cells. Likewise, the highest concentration of HTE20N could decrease 54% of LigB4 and 51% of LigB12 binding to WI-38 cells. These results suggest that binding of LigB to human lung cells may, to some extent, be due to interactions with cell surface tropoelastin.

### HTE17-20 and HTE20N block the adhesion of LigA-expressing *Leptospira* to human lung cells

To investigate whether *Leptospira* attachment to human cells was mediated by LigB-HTE interaction, LigA-expressing *L. biflexa* (Patoc *ligA*) was used for cell adhesion assays, for two reasons. First, this ligA knock-in strain has the ability to adhere to canine kidney (MDCK) cells (Figueira et al., 2011). Second, the HTE-binding domains LigB4 is also present in the conserved region of LigA. In fact, LigB4 shares 100% sequence identity to LigA4. Unfortunately, LigB-expressing *L. biflexa* (Patoc *ligB*) could not serve as a good model for binding assay because it fails to produce full-length LigB protein (Figueira et al., 2011). A serial dilution of Patoc *ligA* was applied to WI-38 cell monolayers, and the binding of the spirochetes to the cells was measured by anti- *L. biflexa* polyclonal antibodies. The antibodies from hamster sera could specifically recognize *L. biflexa* with sound titers (Supplementary Figure 4). Wild-type *L. biflexa* was also included in the study as a negative control. As expected, wild-type *L. biflexa* showed nearly no binding to the WI-38 cells, while Patoc *ligA* displayed significant binding to the cells in a dose-dependent manner (Supplementary Figure 5). To reveal whether the adhesion of Patoc *ligA* to WI-38 cells was impacted by the interaction of surface expressing Lig proteins and HTE, we pre-incubated Patoc *ligA* with exogenous HTE17-20 and HTE20N prior to the addition to WI-38 cells. The HTE20C treated Patoc *ligA* was also included in the same experimental setting. As expected, HTE20C could not inhibit the spirochetes binding to human lung cells at any significant level (Figure 7B). In contrast, both HTE17-20 and HTE20N could partially diminish the bacterial adhesion to WI-38 cells. The binding level of the spirochetes to the cells in the absence of exogenous HTE was normalized as 100% adhesion rate. Based on this normalization, the highest concentration of HTE17-20 could block 61% of spirochetes binding to the cells. Similarly, the highest concentration of HTE20N could inhibit 58% of spirochetes binding to the cells. The inhibition induced by HTE17-20 or HTE20 was significantly stronger than that induced by HTE20C (*p* < 0.05). Overall, these findings indicated that the Lig-HTE interaction contributes to the adhesion of *Leptospira* to human lung cells. Furthermore, the recombinant HTE17-20 and HTE20, with further optimization, might be potentially utilized as adhesion blockers to diminish the attachment of *Leptospira* to human lung cells.

## Discussion

Bacterial MSCRAMMs, functioning as adhesion molecules, play an important role in pathogenesis of bacterial diseases. A wide variety of pathogenic bacteria, such as *Staphylococcus* (Schwarz-Linek et al., 2004a,b; Vazquez et al., 2011), *Mycobacteria* (Kuo et al., 2012), *Borrelia* (Coburn et al., 2013), and *Leptospira* (Lin and Chang, 2007; Lin et al., 2009, 2010) have developed several different kinds of surface adhesins to attach to the host cells by interacting ECM molecules including fibronectin, collagen, and elastin. The strong interactions between bacterial adhesins and host ECM have been thought to allow the pathogens to resist mechanical force and further establish successful colonization (Kline et al., 2009). It is likely that *Leptospira* could utilize similar strategy to adhere to broken skin where ECM is highly populated and to reach target organs (e.g., lungs) where ECM is abundant as well. In the severe form of leptospirosis, infected patients could present life-threatening pulmonary hemorrhage, which might be related to the ability of *Leptospira* binding to lung elastin. As previous research demonstrated, LigB might mediate the leptospiral attachment to host cell surface by binding to exons 17th to 27th of HTE (HTE17-27; Lin et al., 2009). HTE is composed of distinct domain modules: Gly-rich hydrophobic domains, Pro-rich hydrophobic domains and crosslinking domains. LigB Ig-like repeats preferentially bound to central region of HTE, suggesting it is likely that the binding sites can be associated with a specific type of domains.

To pinpoint the minimal binding sites for LigB on HTE, we further truncated the central region of HTE into three smaller constructs, each of which contains alternating crosslinking domains and hydrophobic domains. We found that both LigB4 and LigB12 preferentially bound to HTE17-20, the only construct that encompasses two Pro-rich hydrophobic domains. As expected, both LigB repeats specifically recognized HTE20, and to a lesser extent, HTE18, while both repeats had no affinity to crosslinking domains HTE17 and HTE19 (Figure 2). To be noted, the binding level of LigB to HTE20 was only slightly lower than that to HTE17-20 and almost the same as that to HTE19-20 (p > 0.1). This suggests that HTE20 is the main binding site for LigB Ig-like repeats. The small angle X-ray scattering revealed that full-length HTE is an asymmetric molecule, consisting of a N-terminal elongated, rod-like coil region (exons 2–19), a protruded hinge region (exons 20–24), and a branching point (exons 25–26) bridged the main body of HTE with the C-terminal foot region (exons 27–36; Baldock et al., 2011; Yeo et al., 2012). On the basis of the solution structure of HTE and the unique locations of lysine residues on exons 10, 19, and 25, a head-to-tail model for the assembly of monomeric HTE was proposed by Baldock et al. Interestingly, exon 20 (HTE20) seems to remain accessible to the environment in polymeric HTE (Baldock et al., 2011). In agreement with our previous and current findings, LigB Ig-like repeats, specifically bound to HTE20, also interacted with full-length HTE and polymeric elastin (Lin et al., 2009). This might further promote the invasion of pathogenic *Leptospira* during the process of infection.

To establish successful attachment to host tissues, bacterial pathogens have developed a variety of surface adhesins to recognize tropoelastin or elastin. For example, Fibronectin-Binding Protein A (FnBPA) from *Staphylococcus aureus* bound to HTE2-18, HTE17-27 and HTE27-36 (Keane et al., 2007a). Similarly, Antigen 85 complex (Ag85) from *Mycobacterium tuberculosis* also recognized all three different regions of HTE (Kuo et al., 2013). In both cases, it appears that multiple sites from HTE can be targeted by bacterial MSCRAMMs. Provided that crosslinking domains of HTE contains high density of positively charged residues, bacterial adhesins are thought to utilize the surface Asp or Glu to electrostatically bind to HTE. On the other hand, LigB specifically recognized Pro-rich hydrophobic domains instead of crosslinking domains (Figure 2). This indicates the LigB-HTE interaction might depend on distinct mechanism as opposed to other bacterial adhesins.

To gain more insight into the interaction between LigB repeats and HTE truncates, LigB12 was chosen to investigate the real time binding kinetics to HTE17-20, HTE19-20, and HTE20 by SPR (Supplementary Figure 1). HTE17-20 exhibited greatest affinity to LigB12 (*K*_*D*_ = 0.85 ± 0.04 μM), while HTE19-20 and HTE20 showed slightly weaker affinities to LigB (HTE19-20, *K*_*D*_ = 1.34 ± 0.06 μM; HTE20, *K*_*D*_ = 0.94 ± 0.04 μM). The smaller HTE constructs (HTE19-20 and HTE20) dissociated from LigB12 faster than HTE17-20; in other words, larger *k*_*off*_ could be attributed to the weaker LigB-HTE interaction. This also suggests that HTE18 could partially contribute to the binding to LigB. The *k*_*on*_ of the HTE-LigB interactions is not considered as a fast association (~10^3^ M^−1^ s^−1^), but it is comparable to the binding of IgG to Fcg receptor (Li et al., 2007). Furthermore, we used steady state fluorescence spectroscopy to characterize the HTE20-LigB12 interaction. Consistent with the *K*_*D*_ measured by SPR, *K*_*D*_ of HTE20 binding to LigB12 was 0.97 ± 0.03 μM. This submicromolar affinity is comparable to the affinity of HTE27-28 binding to Ag85 (Kuo et al., 2013). Therefore, it is likely that the HTE-LigB interaction is physiological relevant, and HTE20 might potentially serve as adhesion blocker to diminish the leptospiral attachment to the host cell surface. Each individual Ig-like repeat of LigB contains only one tryptophan, which is tightly buried in the hydrophobic core as revealed by high resolution structure of LigB12 (Ptak et al., 2014). The spectrum of LigB12 presented a characteristic doublet maximum in 315 and 326 nm, while the fluorescence intensities of these two peaks were extensively quenched by adding HTE20. This suggests that HTE20 binding site on LigB12 might be in the close proximity of core tryptophan.

Among the hydrophobic domains of HTE, HTE20 is the longest domain containing unique basic and aromatic residues. Sequence alignment of HTE18 and HTE20 showed that these residues are mainly located at N terminal half of HTE20 (HTE20N). In contrast, the C-terminal half of HTE20 (HTE20C) is composed of VPGVG repeats, which is similar to other hydrophobic domains including HTE18. Interestingly, both LigB4 and LigB12 specifically bound to HTE20N but not HTE20C (Figure 3). The affinity of HTE20N to LigB12 measured by SPR and fluorescence spectroscopy was comparable to that to HTE17-20 (SPR, *K*_*D*_ = 0.78 ± 0.05 μM; fluorescence spectroscopy, *K*_*D*_ = 0.82 ± 0.06 μM). Based on these findings, we hypothesized that the residues R360, Y371, F378, and F381 on HTE20N might play roles in binding to LigB. As a result, alanine substitutions of these specific residues all abolished the binding of HTE20 mutants to LigB4 or LigB12 (Figure 4). Overall, we identified that HTE20N is the minimal binding site for LigB, and the positively charged and aromatic residues on HTE20N are critical for this interaction.

According to the high resolution structure of LigB12 that we solved by NMR, single leptospiral Ig-like domain is mainly composed of three β-sheets oriented toward three different surfaces (Ptak et al., 2014). Each surface encompasses around 1000~2000 Å solvent accessible area (calculated by UCSF Chimera; Pettersen et al., 2004), which is large enough to make a complete binding surface for protein binding partner (Chen et al., 2013). To identify the HTE binding sites on LigB, we rationally designed a series of chimeric LigB based on the structural information of LigB12. In each construct of chimera, one or two β-sheets (strands A–C, strands C'–F, and/or strands G–G') from LigB12 was swapped with corresponding β-sheets from LigB5 which had no affinity to HTE. These chimeras maintained the overall structural integrity (examined by CD, data not shown), suggesting that local conformation might also be well-folded to preserve the HTE binding sites on LigB. Intriguingly, the results showed that only the constructs (LigB12-B5-B5, LigB12-B12-B5, and LigB12-B5-B12) containing N terminal β-sheet (strands A-C) of LigB12 retained the ability to interact with HTE (Figure 5). Given that LigB7 could bind to HTE but not LigB10, we also tested the ability of LigB7/B10 chimeras to bind to HTE. Surprisingly, the HTE binding site on LigB7 is also located at the same position, strands A–C (Supplementary Figure 2). These findings suggest there might be a common motif on strands A–C of Ig-like domain, which is responsible for interacting with basic and aromatic residues on HTE20.

Sequence alignment of LigB5, LigB7, LigB10, and LigB12 gave a hint on potential HTE-interacting region within strands A–C. In this potential binding region, two highly conserved negatively charged residues (D1061 and D1066 of LigB12), one hydrophobic residue (F1054) and one small amino acid (A1065) were selected for making site-directed mutagenesis. The binding of D1061N or D1066A mutants to HTE was largely reduced by more than 5 times compared to wild-type LigB12, while the binding of F1054A and A1065K mutants to HTE was fully abolished. Intriguingly, F1054, A1065, D1061, and D1066 are in steric proximity and appear to form a complete binding surface (Figure 6C). In agreement with the fluorescence quenching we observed in Supplementary Figures 1D, 3D, this HTE binding surface is truly close to the core tryptophan (W1073) of LigB12. Furthermore, the interaction between wild-type HTE and LigB12 was diminished when the buffer pH was more than 8 or NaCl concentration was above 300 mM (data not shown). Taken together, it is highly likely that these acidic and hydrophobic residues within the strands A–C of LigB12 are the critical hot spots for binding to HTE through electrostatic and hydrophobic interactions.

In recent severe cases of human leptospirosis, pulmonary hemorrhage has been considered as a major complication leading to lethality. Researchers suggested that the pathogenesis of leptospirosis-associated severe pulmonary hemorrhage syndrome (SPHS) might be distinct from Weil's disease (McBride et al., 2005; Hashimoto et al., 2013). In addition, high leptospiral burdens could be found in the lungs of SPHS patients, and different types of cells in leptospirotic lungs exhibited strong bacterial antigenicity (De Brito et al., 2013). Given that elastin is one of the main ECM components in lungs, it is likely that Lig proteins play a role in mediating leptospiral attachment to the host cells through binding to lung elastin. To this end, we examined if the recombinant Lig proteins and Lig protein-expressing spirochetes could adhere to an elastin- secreting WI-38 cells, human embryonic lung fibroblasts. The results showed that either Lig proteins produced in the recombinant form or displayed on the bacterial surface could bind to WI-38 cells. Moreover, this pathogen-host interaction could be largely inhibited in the presence of recombinant HTE17-20 or HTE20N (Figure 7). Although, the highest concentration (10 μM) of exogenous HTE could not fully abolish the binding of Lig proteins or spirochetes to lung cells. In contrast, 3.5 μM of HTE could almost completely block the interaction of Lig proteins with immobilized HTE (Lin et al., 2009). This indicates that Lig proteins, besides recognizing elastin, might also interact with other receptors on the lung cells. It is believed that LigA and LigB work complementary to mediate the adhesion of *Leptospira* to host cells. *ligB* knock-out strain still retained the ability to bind to MDCK cells (Croda et al., 2008), which suggests that only targeting one Lig protein would be inefficient for reducing the virulence of *Leptospira*. Here, we identified a small region of HTE could not only recognize conserved region of LigA and LigB, but also block the binding of LigA-expressing spirochetes to mammalian cells. To be noted, pathogenic *Leptospira* harbors other HTE binding proteins which could also mediate the bacterial attachment to the host cells. We will examine whether the HTE fragments identified in this study can also block the adhesion of pathogenic *Leptospira* to lung cells. Moreover, the conserved binding sites of HTE targeted by different *Leptospira* adhesins will be investigated.

In conclusion, we narrowed down the Lig proteins binding sites on HTE to a single Pro-rich hydrophobic domain (HTE20) and identified the key LigB-interacting residues R360, Y371, F378, and F381 on HTE20N. A common motif, DXXX(A/S)D on strands A–C of Lig proteins could compose a complete interface to associate with HTE. In addition, both surface charge-charge and hydrophobic interactions play a role in Lig-HTE interaction. Excessive amount of HTE might have potential to serve as a blocker to diminish the bacterial adhesion to lung cells. This is the report that deciphers the interaction between a leptospiral surface protein and ECM in structural detail. Future work will continuously focus on identifying the atomic resolution of this pathogen-host binding interface.

## Author contributions

Designed the experiments: CH, YC. Performed the experiments: CH, AT. Analyzed the data: CH, AT, YC. Contributed reagents/materials/analysis tools: HH, CK, XW. Wrote the paper: CH, YC.

## Funding

This work was supported in part by the Technology Foundation (CAT) Biotechnology Research (CAT) and Development Corporation (BRDC).

### Conflict of interest statement

The authors declare that the research was conducted in the absence of any commercial or financial relationships that could be construed as a potential conflict of interest.
