# Supplementary material for: Leptospira Immunoglobulin-Like Protein B Interacts with the 20th Exon of Human Tropoelastin Contributing to Leptospiral Adhesion to Human Lung Cells
Source: Front Cell Infect Microbiol. 2017 May 9;7:163. doi: 10.3389/fcimb.2017.00163 (PMC5422739; doi:10.3389/fcimb.2017.00163)

Supplementary Material

Supplementary Table 1. Oligonucleotides used for generating LigB and HTE truncations (Restriction enzyme sites are underlined)

Primer ID	Sequence (5' → 3')
LigB4 fp	CGGAATTCACTCCAGCAGCCTTA
LigB4 rp	CCGCTCGAGCTACAAAGCAGCTTGTGTAAC
LigB5 fp	CGCGGATCCACACAAGCTGCTTTG
LigB5 rp	CCCAAGCTTCTAGAGAACCGCAGGAAC
LigB7 fp	CGCGGATCCACAGCTGCAAAGCTT
LigB7 rp	CCGCTCGAGCTACAATTGTGCCGGAGTTAC
LigB10 fp	CGCGGATCCACTGACTTAAAACTG
LigB10 rp	CCCAAGCTTCTATAACGTGGCAGCACT
LigB12 fp	CGCGGATCCGCAGCAACCCTTTCT
LigB12rp	CCCAAGCTTCTACGTGTCCGTTTTGTT
HTE17-20 fp	CCGGAATTCGGCGTTGGGACTCCA
HTE17-20 rp	CCGCTCGAGCTAGGAAATGCCAACTCC
HTE21-24 fp	CCGGAATTCCCCGAAGCTCAGGCA
HTE21-24 rp	CCGCTCGAGCTAAATGCCGGGAGCCAC
HTE25-27 fp	CCGGAATTCGGCCCTGGTGGAGTT
HTE25-27 rp	CCGCTCGAGCTAATATTTGGCTGCTTTAGC
HTE17 rp	CCGCTCGAGCTAATACTTGGCTGCCTT
HTE18 fp	CCGGAATTCGGAGCTGCTGCAGGC
HTE18 rp	CCGCTCGAGCTATGGAACCGCAGCACC
HTE19 fp	CCGGAATTCGGGGTTGTGTCACCA
HTE19 rp	CCGCTCGAGCTAGTATTTGGCTGCCTT
HTE20 fp	CCGGAATTCGGGGCCAGGCCCGGA
HTE20N rp	CCGCTCGAGCTATCCGACACCAAAGCC
HTE20C fp	CCGGAATTCGTCGGAGGTATCCCT

Supplementary Table 2. Oligonucleotides used for making site-directed mutagenesis (Mutation sites are bold)

Primer ID	Sequence (5' → 3')
HTE20R360A fp	CCGAATTCGGGGCC**GCG**CCCGGAGTCG GAG
HTE20R360A rp	CTCCGACTCCGGG**CGC**GGCCCCGAATTCGG
HTE20Y371A fp	GAGGCATTCCTACT**GCC**GGGGTTGGAGCTG
HTE20Y371A rp	CAGCTCCAACCCC**GGC**AGTAGGAATGCCTC
HTE20F378A fp	TTGGAGCTGGGGGC**GCT**CCCGGCTTTGGTG
HTE20F378A rp	CACCAAAGCCGGG**AGC**GCCCCCAGCTCCAA
HTE20F381A fp	GGGCTTTCCCGGC**GCT**GGTGTCGGAGTC
HTE20F381A rp	GACTCCGACACC**AGC**GCCGGGAAAGCCC
LigB12F1054A fp	CCGTATCAAAACAATTC**GCC**GCGGTGGGAACGTATT
LigB12F1054A rp	AATACGTTCCCACCGC**GGC**GAATTGTTTTGATACGG
LigB12D1061N fp	GTGGGAACGTATTCG**AAT**GGAACCAAAGCGGAT
LigB12D1061N rp	ATCCGCTTTGGTTCC**ATT**CGAATACGTTCCCAC
LigB12A1065K fp	TCGGATGGAACCAAA**AAG**GATTTAACTTCTTCGG
LigB12A1065K rp	CCGAAGAAGTTAAATC**CTT**TTTGGTTCCATCCGA
LigB12D1066A fp	GATGGAACCAAAGCG**GCT**TTAACTTCTTCGGTTAC
LigB12D1066A rp	GTAACCGAAGAAGTTAA**AGC**CGCTTTGGTTCCATC
LigB12E1088A fp	GTGAGTAACGCATCT**GCA**ACGAAAGGATTGGTT
LigB12E1088A rp	AACCAATCCTTTCGT**TGC**AGATGCGTTACTCAC

Supplementary Table 3. Oligonucleotides used for generating LigB chimeras

Primer ID	Sequence (5’ → 3’)
B7/B10/1	GAAATATCCGCAGCCATTTACAATTCTATAAGCAG
B7/B10/2	CTGCTTATAGAATTGTAAATGGCTGCGGATATTTC
B7/B10/3	CCGATATTACAAATCAAGTGACCTGGTATTCTTC
B7/B10/4	GAAGAATACCAGGTCACTTGATTTGTAATATCGG
B7/B10/5	CAAGAAATTACGAATCTTGTTACTTGGAATTCCTC
B7/B10/6	GAGGAATTCCAAGTAACAAGATTCGTAATTTCTTG
B7/B10/7	TCCGACATCTATGCGCTCGGTTCAATCAAAAG
B7/B10/8	CTTTTGATTGAACCGAGCGCATAGATGTCGGA
B5/B12/1	ACGATTACCGCAACCTACGGTTCAGTATCTG
B5/B12/2	CAGATACTGAACCGTAGGTTGCGGTAATCGT
B5/B12/3	GGATATTACGGATCAAGTTACATGGTCCAGC
B5/B12/4	GCTGGACCATGTAACTTGATCCGTAATATCC
B5/B12/5	GGATATTACGGATCAAGTTACATGGTCCAGC
B5/B12/6	AGAATTCCAAGTGACCGAAGAAGTTAAATCC
B5/B12/7	ATAATCACAGCGACCTTAGGAAAAGTTGCAG
B5/B12/8	CTGCAACTTTTCCTAAGGTCGCTGTGATTAT

Supplementary Figure 1. LigB12 interacts with HTE17-20, HTE19-20 and HTE20 with submicromolar binding affinities. **A-C**. SPR analysis of LigB12-HTE interactions were conducted by flowing various concentrations of (A) HTE17-20 (B) HTE19-20 and (C) HTE20 at 0, 0.16, 0.32, 0.63, 1.25, 2.5 and 5μM through LigB12-immobilized chip. The sensogram shown in each panel was a representation of one of the three independent experiments. The K_D, k_{on}, and k_{off} values of these interactions were shown in Table 1 and they were obtained from the average of these three experiments. **D**. A series of two-fold dilutions of HTE20 (10 μM ~ 0.625 μM) were gradually titrated into LigB12 (2μM), and the fluorescence intensity of LigB12 core Tryptophan was individually recorded at 305~400 nm. The inlet shows the change of fluorescence intensity of various concentrations of LigB-HTE mixtures measured at 315 nm and plotted as a function of HTE20 concentrations. The saturation curve was then fitted with the equation mentioned in materials and methods for calculating the dissociation constant (K_D), which is shown in Table 1. Shown was a representation of six experiments performed on three separate occasions.

Supplementary Figure 2. HTE17-20, HTE20 and HTE20N bind to the first β-sheet of LigB7. A-C. The binding of LigB7/LigB10 chimeras to (A) HTE17-20, (B) HTE20 or (C) HTE20N was analyzed by ELISA. Sole LigB10 was also included as a negative control [Lin et al. J. Biol. Chem. 2009]. Five micromolar of histidine tagged HTE17-20, HTE20 and HTE20N were applied to LigB7/LigB10 chimeras coated wells (1μM/well). The binding of truncated HTE to Lig proteins was detected by anti-His antibodies. All experiments were conducted in triplicates. Each bar represents the mean of three independent determinations ± 1 standard deviation. Asterisks indicate that binding was significantly different from binding of HTE to the LigB10 ($p < 0.05$, t-test). **D**. Amino acids sequence alignment of HTE-binding Ig-like domains (LigB12, LigB7) and the domains without HTE-binding ability (LigB5, LigB10). The potential residues which might be responsible for binding to HTE are highlighted in red. Asterisk indicates that LigB7 has identical sequence as LigB4 in this region.

Supplementary Figure 3. SDS-PAGE analysis of LigB12 wild-type and mutants. Tag-free LigB12 wild-type (WT) and mutants were purified by Ni-NTA and size exclusion chromatography. Then, the protein samples were subjected to 15% SDS-PAGE analysis and stained with Coomassie blue. Lane 1: pre-stained protein marker; lane 2: WT; lane 3: F1054A; lane 4: D1061N; lane 5: A1065K; lane 6: D1066A; lane 7: E1088A.

Supplementary Figure 4. Polyclonal antibodies from hamster sera specifically recognize *L. biflexa.* Polyclonal antibodies specifically against *L. biflexa* were generated by immunizing hamsters with spirochetes twice tri-weekly. Hamster sera were collected before the first immunization (red circles) and after the second immunization (black squares), and then titers of sera were measured by ELISA. Various concentration of sera (1/500, 1/1000, 1/2000, 1/4000, 1/8000, 1/16000, 1/32000, 1/64000) were applied to *L. biflexa* or BSA (negative control) coated microtiter wells. The binding of polyclonal antibodies to immobilized *L. biflexa* was detected by goat anti-hamster IgG antibodies conjugated with HRP. All experiments were conducted in three independent trials and the

mean ± 1 standard deviation of the results was shown. The binding curves were fit with the function of Logistic using OriginLab software (version 7.0).

Supplementary Figure 5. LigA-expressing *L. biflexa* binds to WI-38 cells in a dose dependent manner. A serial two-fold dilution of *L. biflexa* Patoc *lig*A (black squares) or *L. biflexa* wild-type (WT, red circles) was individually added to overnight-grown WI-38 cells in microtiter plates. The binding of *L. biflexa* to WI-38 cells was measured by ELISA. All experiments were conducted in three independent trials and the mean ± 1 standard deviation of the results was shown. The binding curves were fit with the function of Logistic using OriginLab software (version 7.0). Asterisk indicates that binding of Patoc *lig*A to the cells was significantly greater than WT ($p < 0.05$, t-test).

Supplementary Figure 1

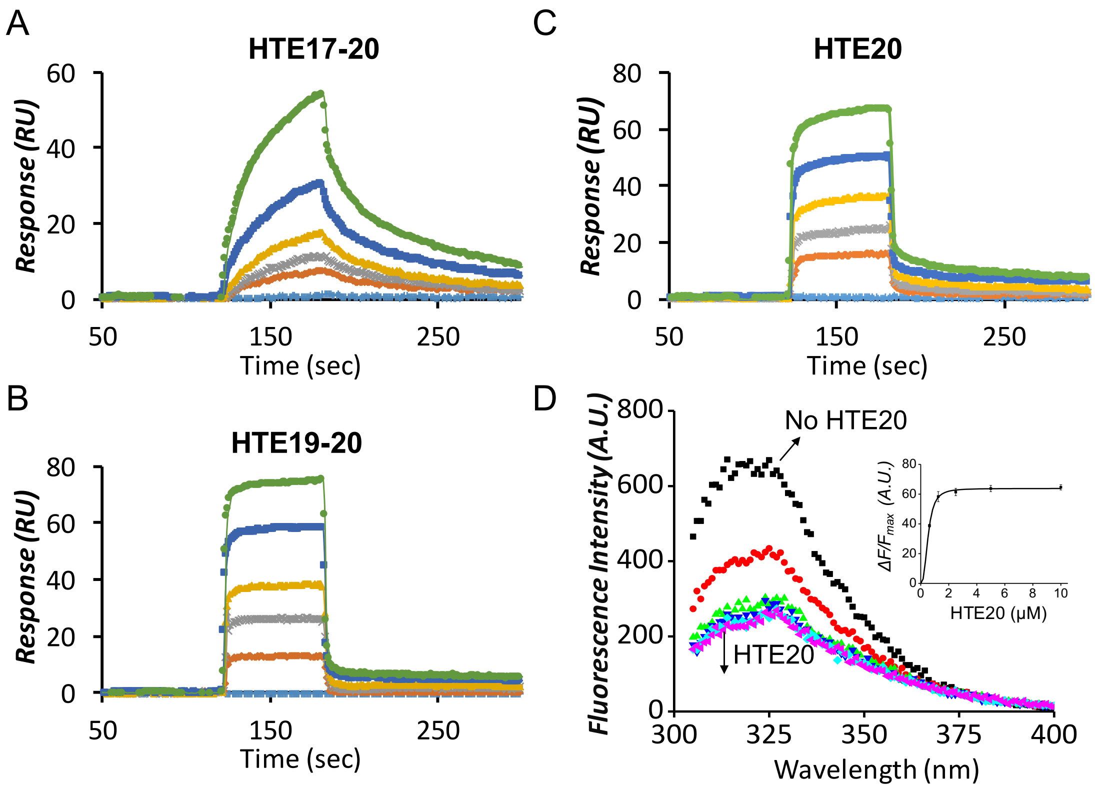

Supplementary Figure 2

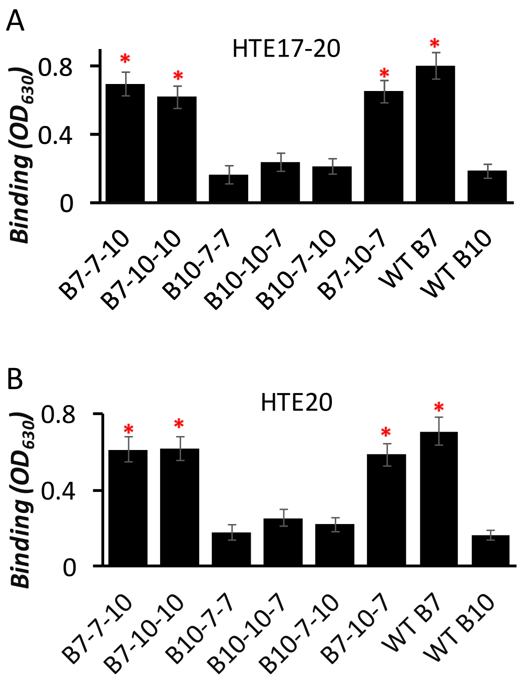

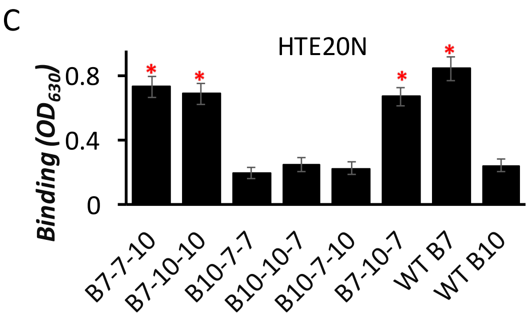

D

LigB12	1061	DGTKADLT	1068
LigB5	431	DNSKKDIT	438
LigB7*	611	DNSNSDIT	618
LigB10	880	DGSEQEIT	887

Supplementary Figure 3

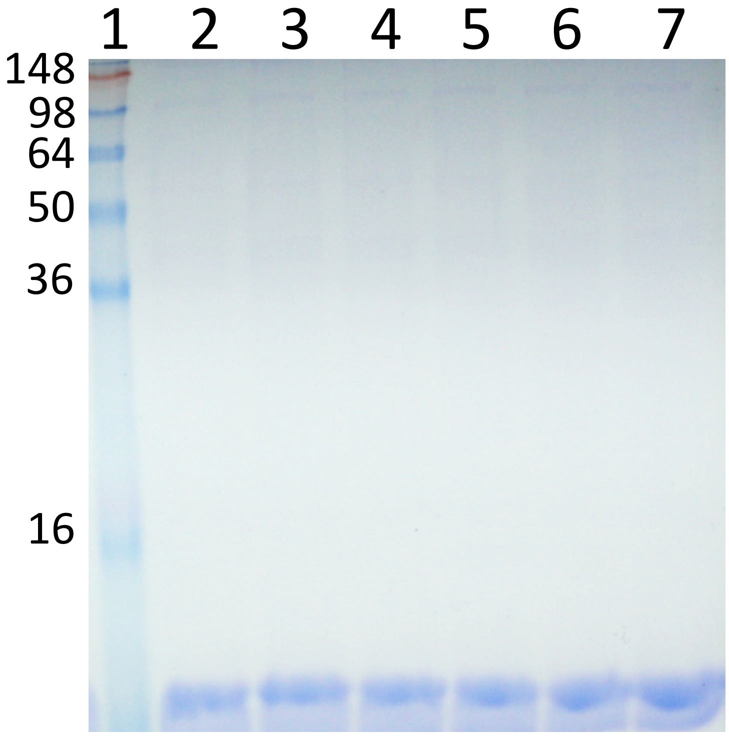

Supplementary Figure 4

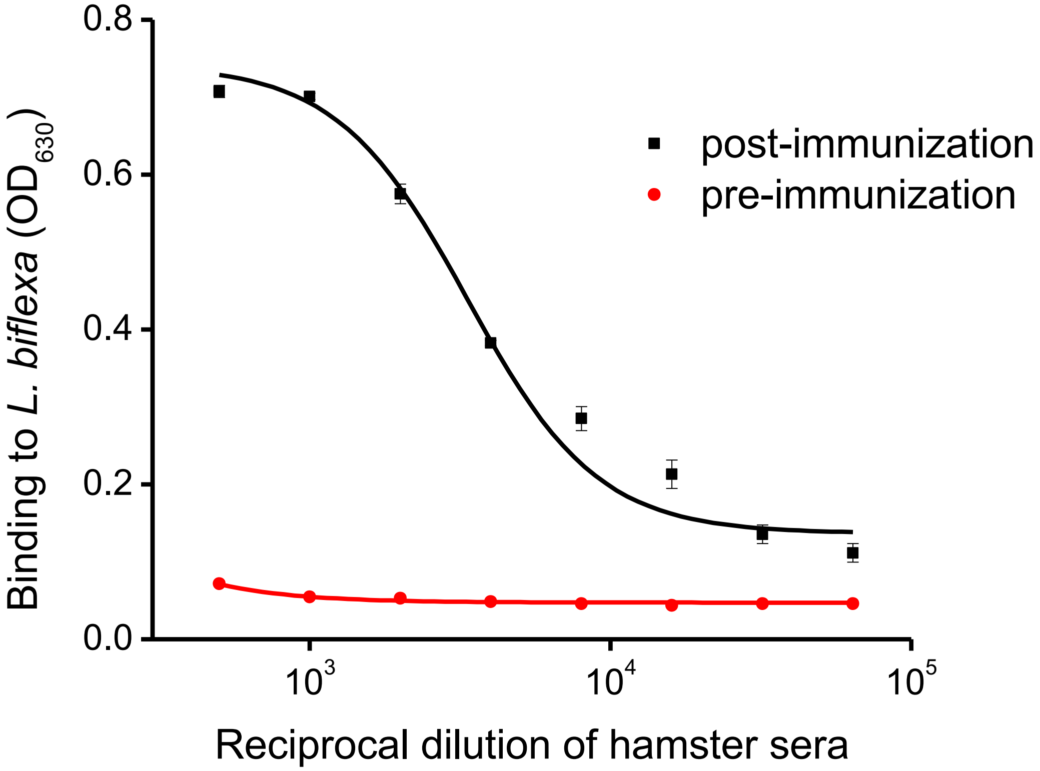

Supplementary Figure 5

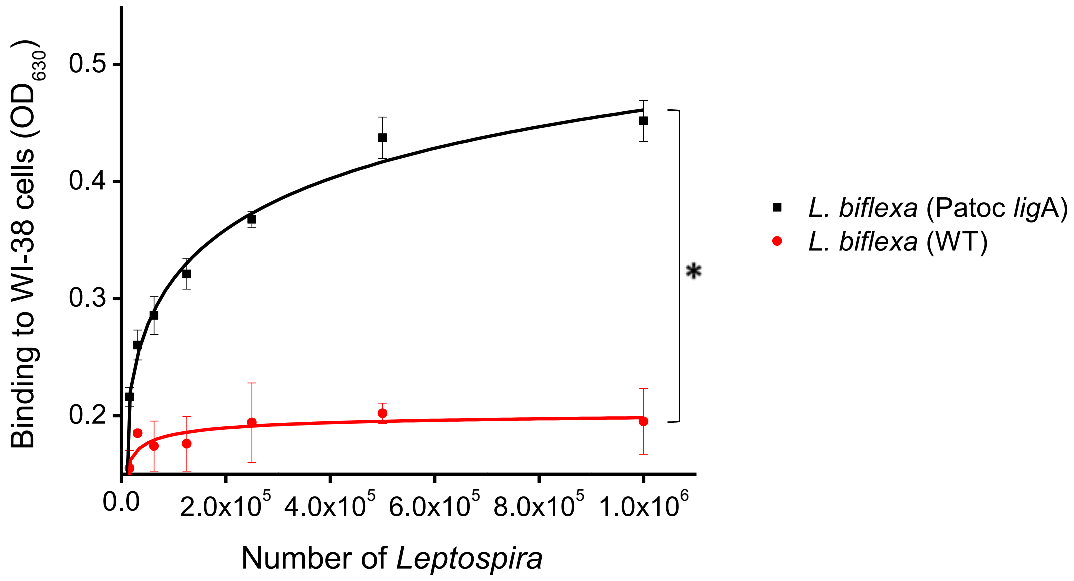

Supplement: Supplementary file 1 [file Table1.PDF]
